# Microbial Community Responses to Organophosphate Substrate Additions in Contaminated Subsurface Sediments

**DOI:** 10.1371/journal.pone.0100383

**Published:** 2014-06-20

**Authors:** Robert J. Martinez, Cindy H. Wu, Melanie J. Beazley, Gary L. Andersen, Mark E. Conrad, Terry C. Hazen, Martial Taillefert, Patricia A. Sobecky

**Affiliations:** 1 Department of Biological Sciences, University of Alabama, Tuscaloosa, Alabama, United States of America; 2 Earth Sciences Division, Lawrence Berkeley National Laboratory, Berkeley, California, United States of America; 3 Department of Civil and Environmental Engineering, University of Tennessee, Knoxville, Tennessee, United States of America; 4 School of Earth and Atmospheric Sciences, Georgia Institute of Technology, Atlanta, Georgia, United States of America; Missouri University of Science and Technology, United States of America

## Abstract

**Background:**

Radionuclide- and heavy metal-contaminated subsurface sediments remain a legacy of Cold War nuclear weapons research and recent nuclear power plant failures. Within such contaminated sediments, remediation activities are necessary to mitigate groundwater contamination. A promising approach makes use of extant microbial communities capable of hydrolyzing organophosphate substrates to promote mineralization of soluble contaminants within deep subsurface environments.

**Methodology/Principal Findings:**

Uranium-contaminated sediments from the U.S. Department of Energy Oak Ridge Field Research Center (ORFRC) Area 2 site were used in slurry experiments to identify microbial communities involved in hydrolysis of 10 mM organophosphate amendments [i.e., glycerol-2-phosphate (G2P) or glycerol-3-phosphate (G3P)] in synthetic groundwater at pH 5.5 and pH 6.8. Following 36 day (G2P) and 20 day (G3P) amended treatments, maximum phosphate (PO_4_^3−^) concentrations of 4.8 mM and 8.9 mM were measured, respectively. Use of the PhyloChip 16S rRNA microarray identified 2,120 archaeal and bacterial taxa representing 46 phyla, 66 classes, 110 orders, and 186 families among all treatments. Measures of archaeal and bacterial richness were lowest under G2P (pH 5.5) treatments and greatest with G3P (pH 6.8) treatments. Members of the phyla *Crenarchaeota*, *Euryarchaeota*, *Bacteroidetes*, and *Proteobacteria* demonstrated the greatest enrichment in response to organophosphate amendments and the OTUs that increased in relative abundance by 2-fold or greater accounted for 9%–50% and 3%–17% of total detected *Archaea* and *Bacteria*, respectively.

**Conclusions/Significance:**

This work provided a characterization of the distinct ORFRC subsurface microbial communities that contributed to increased concentrations of extracellular phosphate via hydrolysis of organophosphate substrate amendments. Within subsurface environments that are not ideal for reductive precipitation of uranium, strategies that harness microbial phosphate metabolism to promote uranium phosphate precipitation could offer an alternative approach for *in situ* sequestration.

## Introduction

Within sediments, the mobility of phosphate (PO_4_^3−^) is controlled by pH, coprecipitation reactions with metals and radionuclides, adsorption/desorption, and ion-exchange reactions [1]. As a result of this poor mobility in subsurface environments, microorganisms release organic acids and/or express phosphatase enzymes (i.e., acid/alkaline phosphohydrolases) to enhance the solubility and cellular transport of phosphate [2]–[4]. Harnessing microbial phosphatases expressed by extant microbial communities within uranium (U)-contaminated environments represents an approach to leverage microbial phosphate acquisition phenotypes to promote *in situ* sequestration of U as insoluble phosphate minerals.

Alternative approaches for microbial mediated U immobilization have examined bioaccumulation, reductive precipitation, ligand-generated precipitation (e.g., carbonate and sulfide), and volatilization reactions to reduce contaminant solubility [5]–[8]. Microbial reductive precipitation of soluble U(VI) to insoluble U(IV) has been extensively examined in both laboratory and field studies where delivery of electron donor substrates, buffered at circumneutral pH, has proven effective as an immobilization strategy [9]–[12]. However, the limitations of U(VI) reduction are observed in environments that experience dynamic geochemical conditions where low pH inhibits microbial U(VI) reduction [11], [13] and reoxidation of U(IV) occurs in the presence of oxygen, nitrate, ferric iron, and humics [14]–[17].

**Figure 1:**
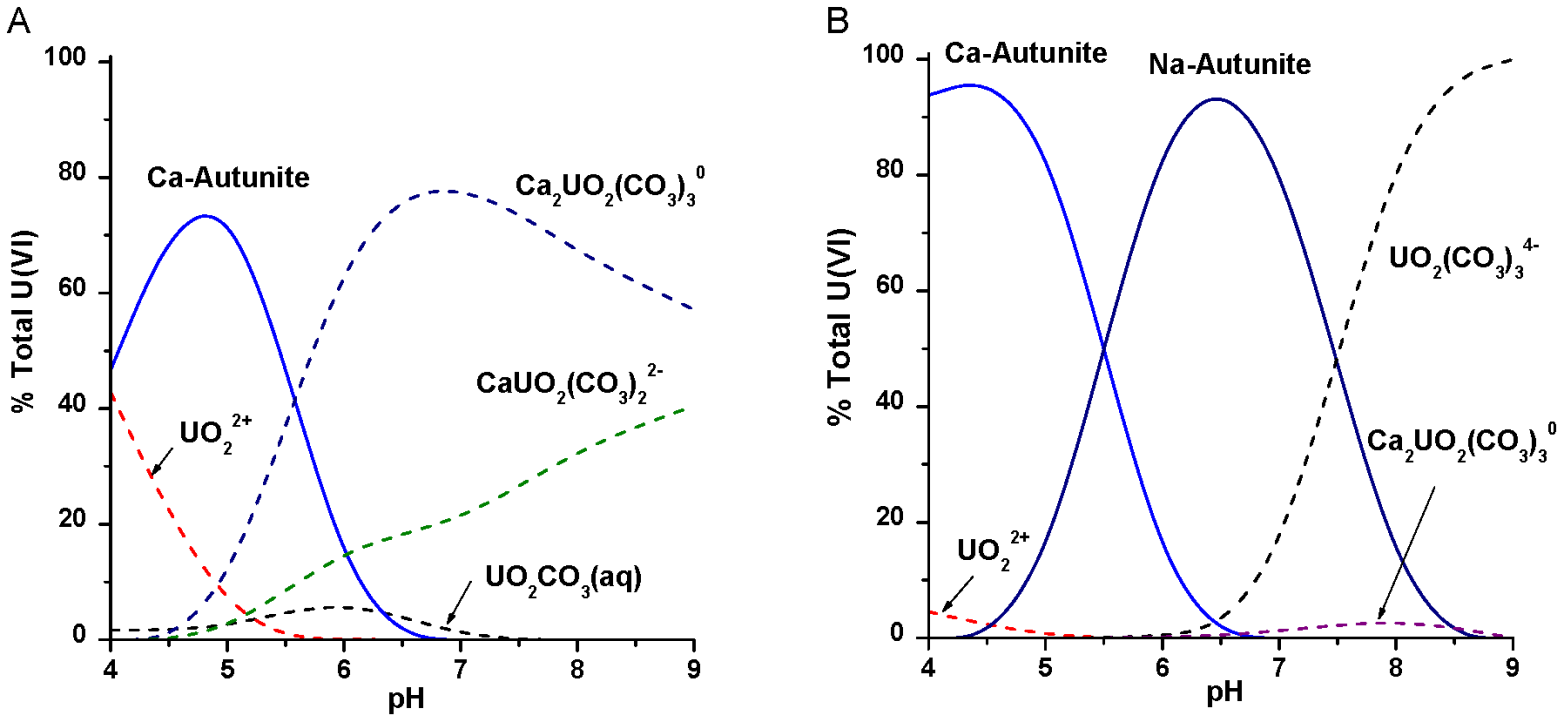
Thermodynamic modeling of U(VI) in the presence of phosphate as a function of pH. ORFRC Area 2 groundwater concentrations of dissolved ions (GW-836 monitoring well), U(VI) = 4.5 µM, Ca^2+^ = 4.85 mM, (A) PO_4_^3−^ = 500 µM and (B) PO_4_^3−^ = 5 mM were used to model the distribution of U(VI) species. Dashed lines represent soluble species and solid lines represent insoluble species.

Within U-contaminated sediments at the United States Department of Energy Oak Ridge Field Research Center (ORFRC), three distinct groundwater contaminant flow paths contribute to pH and co-contaminant heterogeneity (i.e., porewater pH ranging from 3.4–7.0 and [NO_3_^−^] ranging from 29 mg L^−1^ to 2300 mg L^−1^), which inhibit or reverse microbial U(VI) reduction [12], [18]–[20]. Alternatively, *in situ* precipitation of U(VI) as highly insoluble phosphate minerals (e.g., autunite) that remain stable across a broad pH range (Figure 1) offers an approach for U(VI) sequestration under both oxidizing and reducing conditions [21]–[23]. Autunite minerals have been identified in sediments at the U.S. Department of Energy (DOE) Fernald site, Hanford site, and Oak Ridge National Laboratory [24]–[26], suggesting that long-term *in situ* sequestration of U(VI) as phosphate minerals represents a viable remediation strategy. Unfortunately, direct injection of phosphate causes blockage of sediment pore spaces at injection sites due to the rapid precipitation of phosphate with subsurface sediment cations [27]. Therefore, the use of less reactive inorganic or organic phosphate compounds must be employed for effective delivery to deep subsurface contaminated zones where microbial phosphatase activity can hydrolyze these substrates and liberate reactive phosphate.

The activity of phosphatases from several bacterial species [e.g., *Aeromonas*, *Bacillus*, *Myxococcus*, *Pantoea*, *Pseudomonas*, *Rahnella*, and *Serratia* (formerly *Citrobacter* sp.)] have been shown to increase extracellular phosphate concentrations that subsequently promote metal and radionuclide precipitation as highly insoluble mineral phosphates [22], [28]–[33].

Our previous work has shown that organophosphate substrates [i.e., glycerol-3-phosphate (G3P) and glycerol-2-phosphate (G2P)] remain soluble within saturated sediments and in solutions containing uranium [34], [35]. Both G3P and G2P represent organophosphates that are present within sediments: G3P is commonly found in prokaryotic and eukaryotic cell walls, cytoplasm, and lipid membranes [36], [37], while G2P is a less common compound found within bacterial and fungal cell extracts and from phosphatidyl choline alkaline hydrolysis [38]–[40]. Furthermore, our recent ORFRC sediment column studies utilizing both G2P and G3P amendments stimulated the extant microbial community that hydrolyzed both substrates which yielded 1–3 mM phosphate within acidic and circumneutral pH porewater and promoted precipitation of U(VI) [35].

Due to the observed pH and contaminant heterogeneity observed within the ORFRC subsurface, we hypothesized that distinct microbial communities capable of organophosphate hydrolysis would be enriched with G2P or G3P amendments under acidic or circumneutral pH and that hydrolysis of G3P would yield the greatest concentrations of extracellular phosphate. The goal of this study was to utilize the 16S rRNA high-density microarray (PhyloChip), capable of detecting 8,741 archaeal and bacterial taxa [41], to characterize the extant prokaryotic community within ORFRC U-contaminated sediments that contributed to organophosphate (i.e., G2P and G3P) hydrolysis. Due to the heterogeneity of geochemical parameters (pH, [U], [NO_3_^−^], etc) present within the ORFRC subsurface, characterization of extant phosphate solubilizing microbial communities enriched under specific pH and organophosphate amendments can aid in development of strategies for *in situ* phosphate mineralization of U(VI).

## Results

### Microbial response to slurry incubations

**Figure 2:**
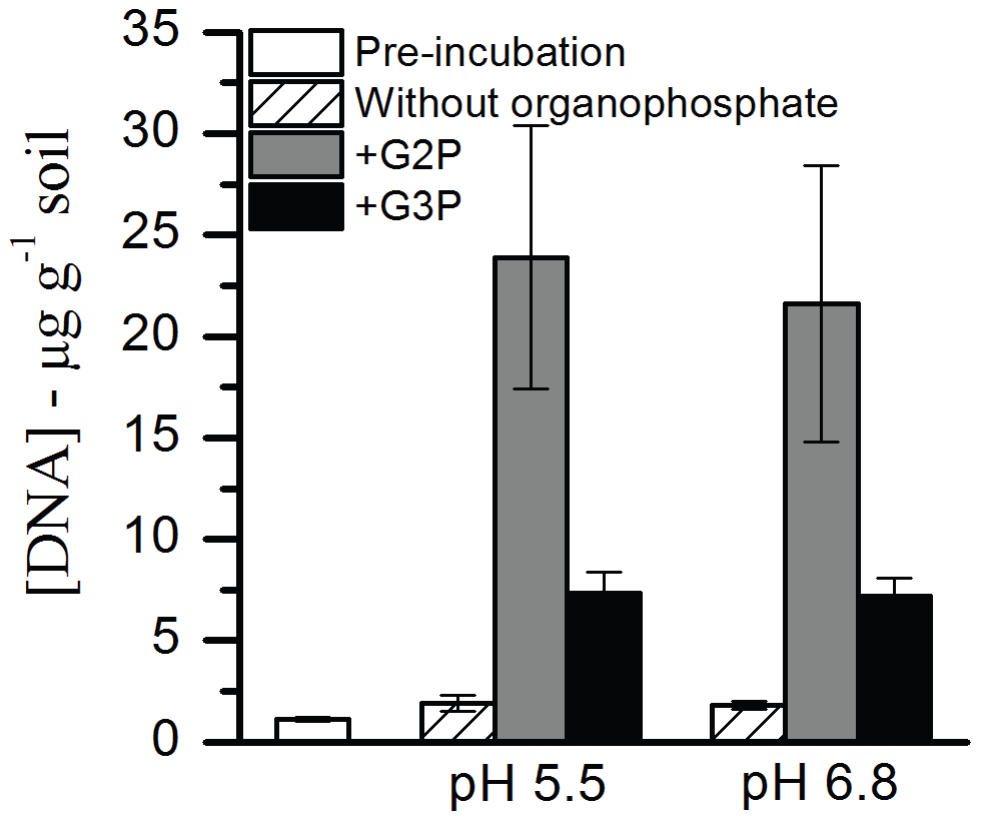
DNA extractions from ORFRC subsurface sediments. DNA concentrations pre- and post-incubations (pH 5.5 and pH 6.8) amended with G2P, G3P, and without organophosphate. Error bars indicate standard deviation of replicate treatments (n = 3).

Total DNA extractions from sediment slurry treatments were measured as a proxy for microbial growth in response to the different incubation conditions. Prior to treatments, ORFRC subsurface sediment DNA concentrations were 1.1±0.1 µg g^−1^ (Figure 2). Sediment slurries incubated at either pH 5.5 or pH 6.8 without organophosphate addition exhibited a 1.7-fold increase (1.8±0.2 to 1.9±0.4 µg g^−1^) in DNA concentration after 36 days. DNA concentrations increased 20-fold (21.6±6.8 to 23.9±6.5 µg g^−1^) after 36 days in G2P-amended treatments and 6-fold (7.2±0.9 to 7.3±1.1 µg g^−1^) after 20 days in G3P-amended treatments at both pH values (Figure 2).

### Chemical analyses of sediment slurry incubations

G2P, G3P, PO_4_^3−^, NO_3_^−^, NO_2_^−^, and organic acids were measured at 96 h intervals over the course of all incubations. Average NO_3_^−^ concentrations among all sediment slurry treatments did not decrease throughout the time course (data not shown) indicating that aerobic conditions were maintained during these incubations.

**Figure 3:**
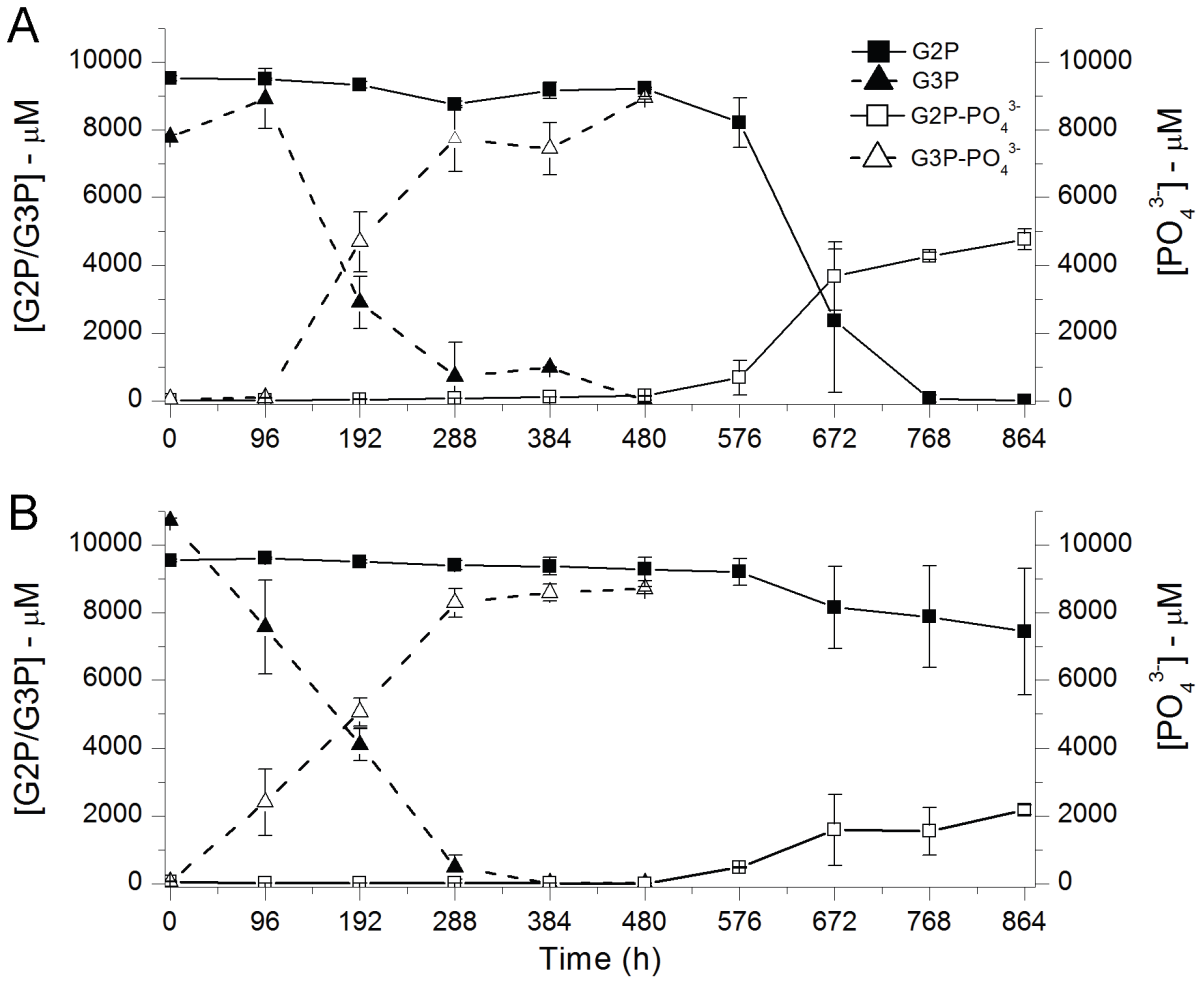
Organophosphate and phosphate measurements. Sediment slurry incubations conducted at (A) pH 5.5 and (B) pH 6.8. Solid lines connect time points in G2P treatments and dashed lines connect time points in G3P treatments.

Phosphate concentrations in the G2P-amended slurries remained below 140 µM for 576 h then increased to 4.8 mM and 2.2 mM in the pH 5.5 and 6.8 incubations, respectively (Figures 3A and 3B). Combined concentrations of G2P and soluble phosphate in the pH 6.8 treatments exhibited that mass balance of PO_4_^3−^ was respected throughout the entire time course (Figure 3B). Conversely, at pH 5.5, G2P was completely removed from solution without a proportional accumulation of soluble phosphate after 576 h (Figure 3A). In contrast to the G2P treatments, G3P was completely consumed within 300–400 h at both pH values (Figures 3A and 3B). Phosphate concentrations in G3P-amended slurries increased after 96 h (pH 5.5) and prior to the 96 h time point (pH 6.8), then accumulated over 4.7 mM phosphate by the 192 h time point. At the 480 h time point, soluble phosphate concentrations reached 8.9 mM and 8.7 mM phosphate in G3P (pH 5.5) and G3P (pH 6.8) treatments, respectively (Figures 3A and 3B).

### Archaeal community structure

**Figure 4:**
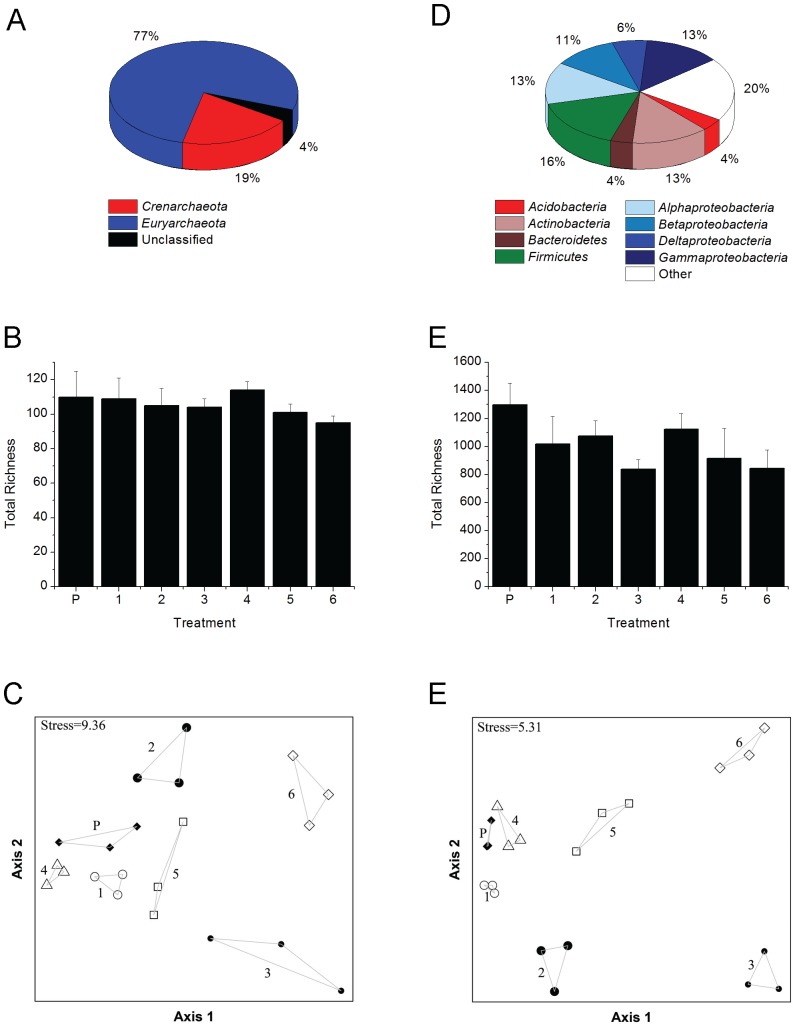
Archaeal and bacterial community structure. (A) Phylum-level richness of *Archaea* detected in sediments prior to treatments. (B) Archaeal richness detected in sediments pre- and post-treatments. (C) NMDS ordination of archaeal community distances present within replicate samples. (D) Phylum-level richness of *Bacteria* detected in sediments prior to treatments. (E) Bacterial richness detected in sediments pre- and post-treatments. (F) NMDS ordination of bacterial community distances present within replicate samples. Designations for all samples are as follows: **P**- sediments prior to treatment, **1**-pH 5.5 without organophosphate amendment, **2**-pH 5.5 amended with G2P, **3**-pH 5.5 amended with G3P, **4**-pH 6.8 without organophosphate amendment, **5**-pH 6.8 amended with G2P, and **6**-pH 6.8 amended with G3P. Phylum-level richness of *Archaea* and *Bacteria* prior to treatments represents the summation of total richness detected from replicate sediment DNA extractions.

A total of 180 archaeal OTUs representing 3 phyla, 10 classes, 16 orders, and 25 families were detected amongst all pre-treatment and treatment samples (Table S1). The phyla *Crenarchaeota* and *Euryarchaeota* accounted for over 96% of the total archaeal richness within ORFRC sediments prior to treatments with the remainder comprised of unclassified *Archaea* (Figure 4A, Table S1). Following treatments, archaeal richness did not change significantly (p-value>0.05) regardless of the amendments (Figure 4B). NMDS ordination of archaeal community composition clustered the replicate samples into distinct groups based on treatment (Figure 4C), and MRPP tests confirmed that archaeal communities differed significantly (δ_o_ = 0.1255, δ_e_ = 0.2798, p-value<0.001, A = 0.5516) amongst all treatments.

**Figure 5:**
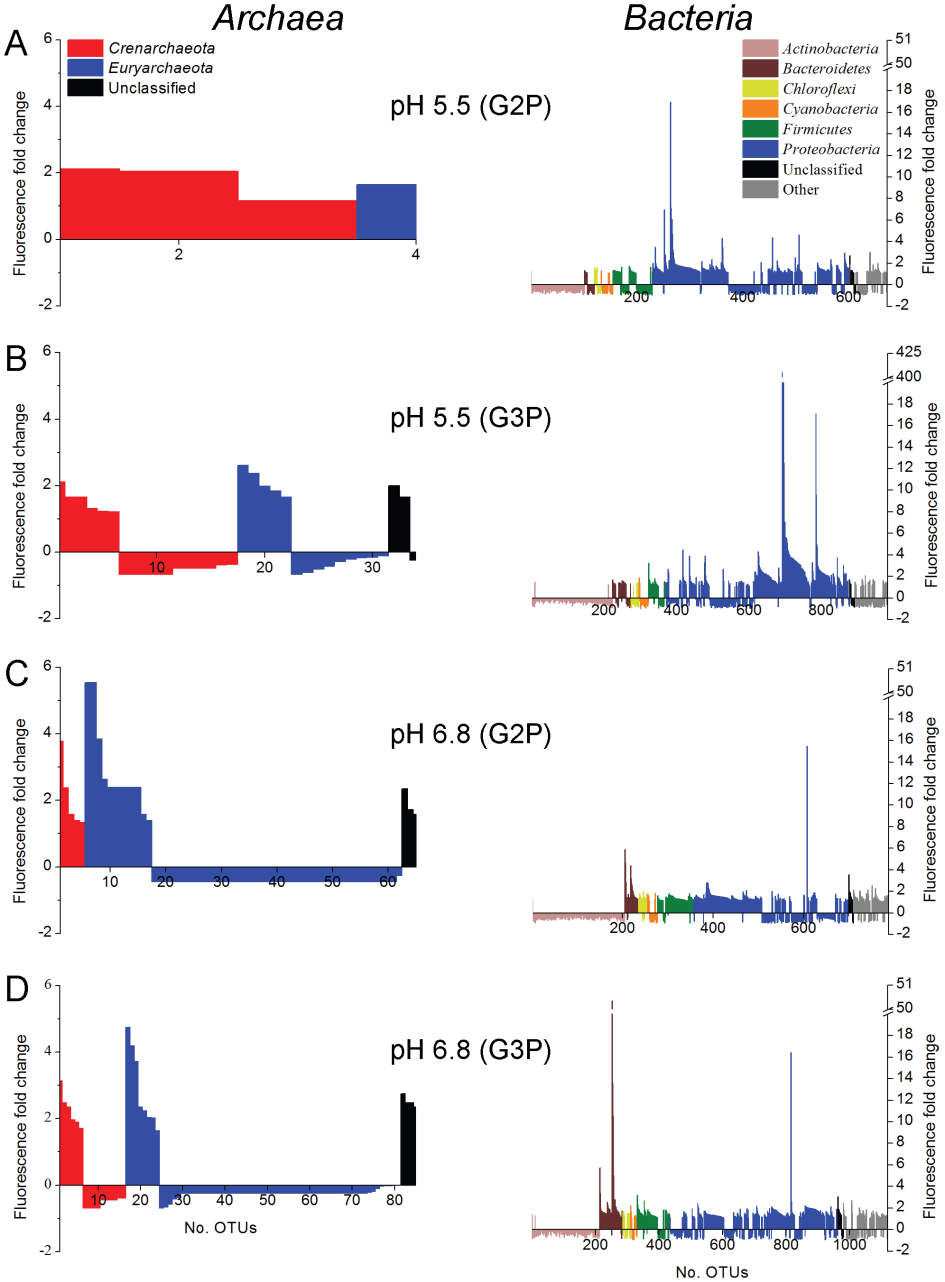
Dynamic archaeal and bacterial OTUs within sediment slurry treatments. Total detected archaeal (left column) and bacterial (right column) OTUs compiled from replicate treatments that significantly increased or decreased relative to incubations lacking organophosphate. Treatment conditions and total number of taxa plotted: (A) G2P (pH 5.5), (B) G3P (pH 5.5), (C) G2P (pH 6.8), and (D) G3P (pH 6.8). OTUs with a 2-fold or greater decrease in fluorescence were not detected.

**Figure 6:**
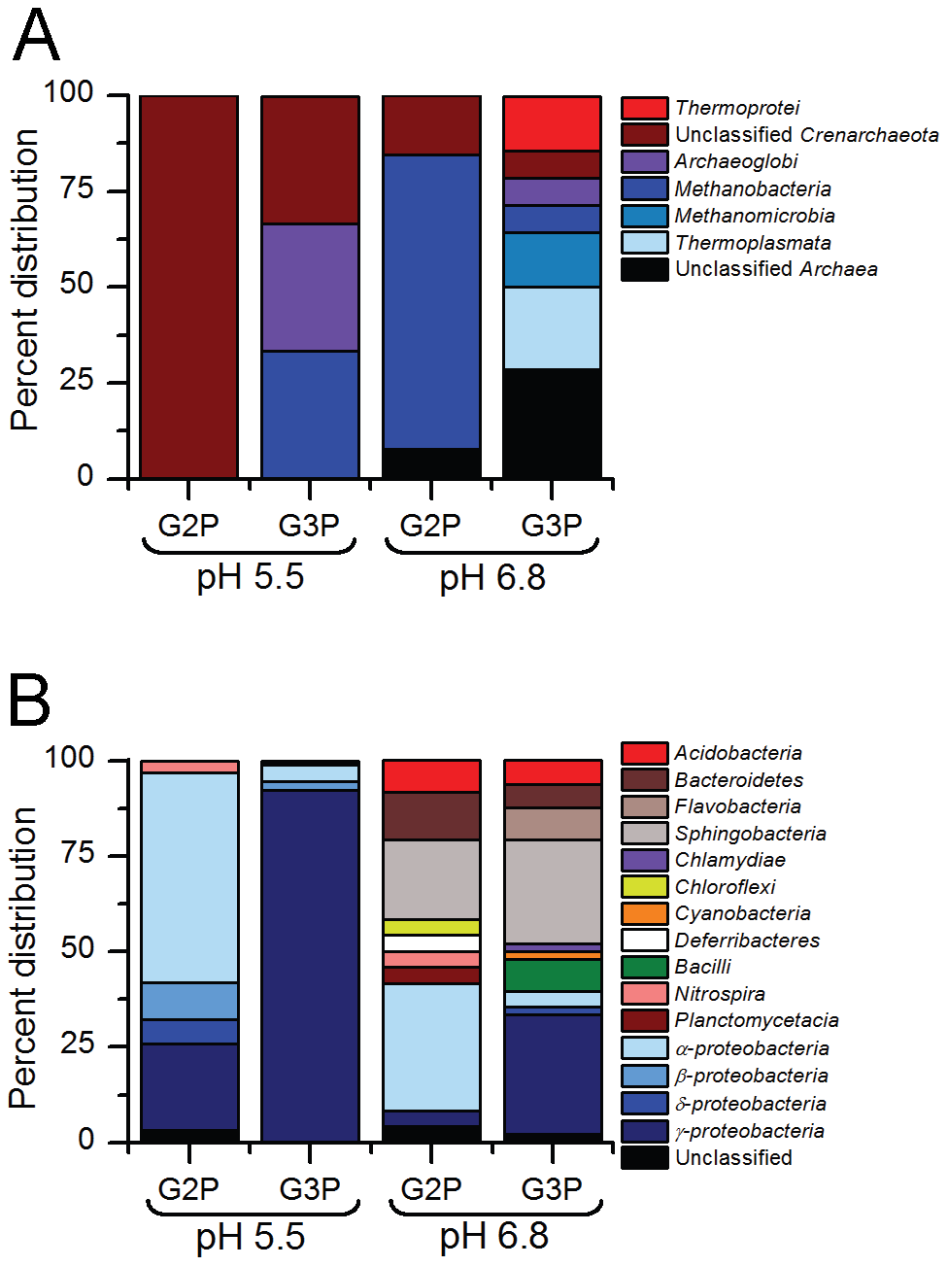
Class-level distribution of enriched OTUs. Compiled OTUs enriched (i.e., 2-fold or greater increase in relative abundance) in replicate treatments representing the most responsive (A) archaeal and (B) bacterial classes from treatments amended with organophosphates at pH 5.5 and 6.8.

The combined influence of pH and organophosphate addition shaped the archaeal community by affecting OTU abundance relative to treatments at the same pH without organophosphate (Figures 5, Tables S1 and S2). Relative to total richness detected in sediments prior to treatments (134 OTUs), the richness of OTUs responding to treatment conditions decreased by 34-fold (4 OTUs), 4-fold (34 OTUs), 2-fold (65 OTUs), and 1.6-fold (85 OTUs) in G2P (pH 5.5), G3P (pH 5.5), G2P (pH 6.8), and G3P (pH 6.8) treatments, respectively (Figure 5). *Archaea* that demonstrated a relative increase in abundance of 2-fold or greater in G2P (pH 5.5) treatments consisted of two unclassified *Crenarchaeota* OTUs. In the G3P (pH 5.5) treatments, one *Crenarchaeota* OTU (unclassified at the class level) and two *Euryarchaeota* OTUs belonging to the classes *Archaeoglobi* and *Methanobacteria* demonstrated a 2-fold or greater increase in abundance (Figure 6A and Table S2). Class-level distribution with a 2-fold or greater increase in abundance in G2P (pH 6.8) treatments contained 13 OTUs composed of unclassified *Crenarchaeota* (15%), *Methanobacteria* (77%), and unclassified *Archaea* (8%). In the G3P (pH 6.8) treatments, 14 OTUs that increased in abundance by 2-fold or greater were composed of *Thermoprotei* (14%), unclassified *Crenarchaeota* (7%), *Archaeoglobi* (7%), *Methanobacteria* (7%), *Methanomicrobia* (14%), *Thermoplasmata* (21%), and unclassified *Archaea* (29%) (Figure 6A and Table S2).

Observed changes in relative abundance were identified in 4 OTUs [G2P (pH 5.5)], 34 OTUs [G3P (pH 5.5)], 65 OTUs [G2P (pH 6.8)], and 85 OTUs [G3P (pH 6.8)]. Four archaeal OTUs belonging to *Archaeoglobi*, *Methanobacteria*, and two unclassified classes of *Crenarchaeota* (related to deep-sea sediment and landfill leachate environmental clones) were detected at a 2-fold or greater increase in relative abundance in multiple treatment conditions (Figure S1A and Table S3). The *Crenarchaeota* (landfill leachate related clone), *Archaeoglobi*, and *Methanobacteria* OTUs detected in multiple treatment conditions [i.e., G3P (pH 5.5), G2P (pH 6.8), and G3P (pH 6.8)] were most abundant in the G3P (pH 6.8) treatments, i.e., 29%–81% greater relative abundance when compared to the other treatments. The second *Crenarchaeota* OTU (deep-sea related clone) was 81% more abundant in treatment conditions with G2P (pH 6.8) relative to G3P (pH 5.5) treatments.

### Bacterial community structure

A total of 1,940 bacterial OTUs, representing 43 phyla, 56 classes, 94 orders, and 161 families, were detected amongst all pre-treatment and treatment samples (Table S1). Prior to incubations, 1540 OTUs representing 42 phyla were identified: 43% belonged to the phylum *Proteobacteria*, 20% belonged to 37 unique phyla, and the remaining 37% consisted of OTUs that belong to the *Acidobacteria*, *Actinobacteria*, *Bacteroidetes*, and *Firmicutes* (Figure 4D, Table S1). G3P treatments at pH 5.5 and pH 6.8 were the only conditions in which a significant decrease (p-value<0.05) in total bacterial richness was observed relative to pre-treatment sediments (Figure 4E). NMDS ordination and MRPP tests confirmed bacterial communities differed significantly (δ_o_ = 0.04862, δ_e_ = 0.137, p-value<0.001, A = 0.6451) based on pre- and post-treatment conditions (Figure 4F). Observed changes in relative abundance were identified in 672 OTUs [G2P (pH 5.5)], 983 OTUs [G3P (pH 5.5)], 788 OTUs [G2P (pH 6.8)], and 1120 OTUs [G3P (pH 6.8)] (Figure 5). Within these treatments, only 3%–17% of detected OTUs increased in relative abundance by 2-fold or greater.

Within the pH 5.5 treatments, the phylum *Proteobacteria* accounted for 94%–99% of the 29 OTUs detected with a 2-fold or greater increase in relative abundance (Figure 6B and Table S2). In treatments with G2P (pH 5.5), α*-proteobacteria* was the dominant class. The orders *Caulobacterales* and *Rhizobiales* accounted for 14% and 24%, respectively, of all proteobacterial OTUs that increased in abundance by as much as 7-fold. Only one OTU from the family *Hyphomicrobiaceae* was enriched in this treatment and demonstrated the greatest increase in relative abundance (17-fold). The remaining β-, δ-, and γ-*proteobacteria* classes were composed of OTUs from the orders *Burkholderiales*, *Rhodocyclales*, *Desulfobacterales*, *Myxococcales*, *Chromatiales*, *Enterobacteriales*, *Pseudomonadales*, *Thiotrichales*, and *Xanthomonadales*.

Treatments amended with G3P (pH 5.5) contained 164 OTUs that increased by 2-fold or greater relative to unamended (control) treatments and were dominated by the class γ-*proteobacteria* (Figure 6B and Table S2). The orders *Enterobacteriales* and *Pseudomonadales* accounted for 39% and 22%, respectively, of all proteobacterial OTUs with a 2-fold or greater increase in relative abundance. Less than 5% of the OTUs from this treatment increased in relative abundance (increases ranged from 13- to 406-fold) and belonged to the genera *Arsenophonus*, *Pseudomonas*, *Pectobacterium*, *Rahnella*, *Photorhabdus*, *Obesumbacterium*, and *Brenneria*. The remaining α-, β-, and γ-*proteobacteria* classes were composed of OTUs (with relative abundance increases between 2- and 4-fold) from the orders *Rhizobiales*, *Rhodobacterales*, *Rhodospirillales*, *Burkholderiales*, *Hydrogenophilales*, *Aeromonadales*, *Alteromonadales*, *Chromatiales*, *Oceanospirillales*, SAR86, *Thiotrichales*, unclassified *(γ-proteobacteria*), *Vibrionales*, and *Xanthomonadales*.

Within the pH 6.8 treatments, a 40%–60% increase in phylum-level richness was detected for OTUs with a 2-fold or greater increase in abundance relative to pH 5.5 treatments. The dominant phyla under growth conditions at pH 6.8 were *Bacteroidetes* and *Proteobacteria*, and accounted for 71%–79% of all OTUs with a 2-fold or greater increase in relative abundance (Figure 6B and Table S2). In treatments amended with G2P, the phylum *Bacteroidetes* was composed of three orders: *Bacteroidales* (38%), *Cytophagales* (25%), and *Sphingobacteriales* (38%). The distribution of *Proteobacteria* consisted of the orders: *Rhizobiales* (78%), *Sphingomonadales* (11%), and *Enterobacteriales* (11%). An OTU from the family *Enterobacteriales* demonstrated the greatest increase in relative abundance (16-fold) under these treatment conditions and members of the *Prevotellaceae*, unclassified *Bacteroidetes*, and one unclassified Bacteria were shown to increase in abundance by as much as 6-fold.

The G3P (pH 6.8) treatment exhibited the greatest number of phyla that had a 2-fold or greater increase in relative abundance (Figure 6B and Table S2). The phylum *Bacteroidetes* was composed of the *Bacteroidales* (10%), *Flavobacteriales* (20%), *Sphingobacteriales* (65%), and unclassified *Bacteroidetes* (5%). The two dominant proteobacterial orders were *Pseudomonadales* (50%) and *Enterobacteriales* (17%). The following orders comprised 11% or less of the remaining proteobacterial richness: *Alteromonadales*, *Rickettsiales*, *Myxococcales*, and *Vibrionales*. Four OTUs from the order *Sphingobacteriales* (unclassified at the family-level) and one *Enterobacteriaceae* OTU demonstrated the greatest increase in relative abundance in this treatment (11- to 50-fold).

Further analysis of all treatment conditions identified 400 bacterial OTUs that were previously below the limit of detection in sediments prior to any treatments (Table S1). Under all treatment conditions, a subset of the previously undetected OTUs (i.e., 125 OTUs representing 3 phyla, 7 classes, 20 orders, and 22 families) were shown to increase in relative abundance by 2-fold or greater (Table S2). A total of 36 OTUs were detected in two or more treatment conditions at a 2-fold or greater increase in abundance relative to unamended treatments, 17 of the 36 OTUs were undetected in sediments prior to treatment (Figure S1B and Table S3). Ten families within the phylum *Proteobacteria* accounted for 75% of all OTUs detected in multiple treatment conditions. The dominant proteobacterial families OTUs detected in multiple treatment conditions, accounting for over 70% of *Proteobacteria*, belonged to *Enterobacteriaceae*, *Phyllobacteriaceae*, *Pseudomonadaceae*, and *Rhizobiaceae*. Two OTUs from the families *Phyllobacteriaceae* and *Pseudomonadaceae* were detected in three of the four organophosphate-amended treatments with a 2-fold or greater increase in relative abundance.

## Discussion

Within U-contaminated subsurface environments, *in situ* sequestration approaches that minimize contaminant transport under dynamic hydrogeological conditions (i.e., pH, O_2_, and co-contaminants) remain a challenge for many of the U.S. DOE legacy sites. This study examined the extant ORFRC prokaryotic community that could promote *in situ* sequestration of U as geochemically stable autunite-type minerals (Figure 1) through hydrolysis of organophosphate substrates (i.e., G2P and G3P). Our previous work has shown that autunite-type minerals, composed of [U]∶[PO_4_^3−^] in a 1∶1 ratio, were formed during U-biomineralization [34]. Thus, characterization of the prokaryotic community that contributes to organophosphate hydrolysis with a concomitant increase in extracellular phosphate concentration is essential in understanding biogeochemical parameters controlling uranium phosphate precipitation. Within the ORFRC, multiple subsurface pathways exist that contribute to contaminant and pH heterogeneity [20], demonstrating the importance of elucidating the dynamic prokaryotic communities that contribute to organophosphate hydrolysis at both acidic and circumneutral pH.

The change in total extractable DNA following organophosphate treatments (Figure 2) was used as a proxy for increased microbial activity that contributed to increased accumulation of extracellular phosphate. The rapid hydrolysis of approximately 90% of total G3P by the end of the 20-day treatment versus hydrolysis of approximately 20%–50% of G2P at the end of the 36-day treatment (Figure 3) likely reflects the predominance of microbial enzymes that can utilize G3P over G2P as a substrate. Conversely, the greater concentrations of total extracted DNA from G2P relative to G3P treatments could reveal enrichment of prokaryotes adapted to organophosphate assimilation rather than rapid hydrolysis.

Characterization of the extant subsurface archaeal and bacterial community as well as the dynamic OTUs responding to growth treatments was determined via PhyloChip 16S rRNA microarray hybridization. Although direct measure of population abundance is not possible with this method, the capability of detecting 10^7^-10^11^ 16S rRNA gene copies [42] supported our goal in characterizing OTUs most responsive (i.e., OTUs that increased 2-fold or greater in relative abundance were designated as responsive) to organophosphate amendments.

Archaeal community characterization within Oak Ridge National Laboratory U-contaminated sediments is currently limited to examination of U- and Hg- contaminated river sediments shown to be dominated by acetate- and hydrogen-dependent methanogens [43] and the enrichment of hydrogen-dependent methanogens following Area 2 subsurface injection of emulsified vegetable oil [44]. While our studies maintained oxic growth conditions, OTUs related to hydrogen-dependent methanogens increased in relative abundance for all treatments except G2P pH 5.5 (Figure 5, Table S1 and S2). Similarly, recent studies have demonstrated metabolic activity of methanogens within oxic environments and suggest related taxa may have expanded ecological functions [45]–[47]. Additionally, OTUs related to thermophilic *Crenarchaeota* and *Euryarchaeota* were detected in all treatments except G2P (pH 5.5). Earlier studies have identified metabolically active thermophilic *Archaea* and *Bacteria* within temperate sediments suggests that thermophiles can occupy an expanded niche but their influence on local geochemistry remains unknown [48]–[50]. The archaeal OTUs that increased by 2-fold or greater in relative abundance (Figures 5 and 6) represent 9%–50% of total archaeal richness detected in ORFRC sediment slurry treatments. Observations of dynamic archaeal taxa within ORFRC sediments highlight the need for future studies that examine functional contributions under oxic growth conditions.

Of the total observed bacterial richness detected in ORFRC sediment slurry treatments, only 3%–17% demonstrated an increase in relative abundance by 2-fold or greater (Figures 5 and 6). Within the pH 5.5 treatments, the phyla *Proteobacteria* represented 94% (G2P) and 98% (G3P) of the enriched OTUs. Alternatively, *Proteobacteria* and *Bacteroidetes* dominated the pH 6.8 treatments, which combined represented 71% (G2P) and 79% (G3P) of enriched OTUs. From culture-dependent studies, isolates belonging to the phyla *Bacteroidetes*, *Firmicutes*, and *Proteobacteria* have been shown to enhance phosphate solubility within the rhizosphere [51], [52]. Use of the PhyloChip has provided an expanded view of bacterial taxa that can contribute to phosphate-cycling within ORFRC sediments.

The lack of mass balance between organophosphate and phosphate concentrations was observed in the G2P (pH 5.5) treatments and the most dynamic OTU (17-fold increase in relative abundance) was related to the genus *Hyphomicrobium*. Members of the family *Hyphomicrobiaceae* are capable of C_1_ metabolism, denitrification, and polyphosphate accumulation [53], [54]. Interestingly, previous work examining ORFRC subsurface microbial communities capable of denitrification have identified *Hyphomicrobium* spp. as an abundant member of Area 2 ORFRC sediments [55], the dominant denitrifying species within Area 1, 2, and 3 ORFRC groundwater [56], and a readily culturable species from ethanol amended Area 2 sediment enrichments [57]. These observations suggest that in addition to the important role of denitrification within ORFRC sediments, *Hyphomicrobium* species could play a role in sequestering extracellular phosphate via intracellular polyphosphate accumulation. Although polyphosphate accumulation could reduce extracellular phosphate concentrations, this physiological response is essential in controlling the cytotoxicity of metals and radionuclides which ultimately can aid in continued denitrification processes.

The γ-*proteobacteria* were shown to be the most dynamic class within G3P (pH 5.5) treatments where OTUs related to the genera *Arsenophonus*, *Brenneria*, *Pseudomonas*, *Obesumbacterium*, *Pectobacterium*, *Rahnella*, and *Photorhabdus* increased from 13-fold to 406-fold. The enrichment of OTUs related to *Pseudomonas* and *Rahnella* is likely due to the previously described phosphate solubilizing activities of related genera isolated from rhizosphere and U-contaminated sediments [22], [31], [51]. The *Obesumbacterium*-related OTU has not been described as a common phosphate solubilizing isolate but characterization of an encoded phytase in *Obesumbacterium proteus* suggests related strains may be capable of organophosphate hydrolysis [58]. The genera *Arsenophonus*, *Photorhabdus*, *Brenneria*, and *Pectobacterium* contain species that have been described as symbionts or plant pathogens but to date have not been shown to enhance phosphate solubilization [59]–[62].

In the G2P (pH 6.8) treatments, the two dominant classes were *Sphingobacteria* and α-*proteobacteria* but an OTU from the family *Enterobacteriaceae* demonstrated the greatest increase in relative abundance (over 16-fold). The *Bacteroidetes* OTUs that were enriched in this treatment were most closely related to rumen and soil isolates capable of phytase activity [63]–[65]. In addition to phytate hydrolysis, the phytase enzyme has been shown to hydrolyze various organophosphate substrates, including G2P [66]. Thus, enrichment of *Bacteroidetes*-related OTUs may also contribute to the hydrolysis of G2P as well as other organophosphate substrates within the ORFRC subsurface.

Enrichment of *Sphingobacteria* and γ-*proteobacteria*.dominated the treatments at pH 6.8 amended with G3P. The same *Enterobacteriaceae* OTU that exhibited the enrichment in the G2P (pH 6.8) treatment also increased 16-fold in relative abundance. *Sphingobacteriales* OTUs that demonstrated the greatest increases in abundance were related to *Bacteroidetes* clones from soil, river, and wastewater samples [67]–[69]. Within G3P (pH 6.8) treatments, a *Sphingobacteriales* OTU that exhibited the greatest increase in relative abundance (50-fold) was related to a clone associated with polyhydroxyalkanoate (PHA)- and polyphosphate (polyP)-accumulating communities from a biological phosphorus removal reactor.

Additional taxa that have not been described as phosphate-solubilizing bacteria were enriched under all amended treatments and may suggest additional ecological functions within sediments that include organophosphate turnover. Within both G2P treatments, the enrichment of OTUs related to *Chloroflexi*, *Deferribacteres*, *Nitrospira*, and *Planctomycetes* were detected. Analysis of the *Candidatus* Nitrospira defluvii and *Isosphaera pallida* genomes reveal that both encode a putative Class C acid phosphatase that could, in theory, contribute to G2P hydrolysis by related *Nitrospira* and *Planctomycetes* OTUs. The lack of studies that examine *Chloroflexi* and *Deferribacteres* organophosphate utilization underline the need for future studies to determine the physiological capacities of related OTUs. The G3P (pH 6.8) treatments were shown to enrich *Cyanobacteria* and *Chlamydiae* OTUs related to *Euglena* chloroplast symbionts and pathogens harbored by and *Acanthamoeba* spp., respectively [70], [71]. Due to the fact that all incubations where conducted in the dark, it is unlikely that photosynthetic algae where enriched but further studies are required to determine if these OTUs were enriched as a result of protozoan-association. Within both pH treatments amended with G3P, six OTUs from the family *Vibrionaceae* were enriched. Although this finding has not been reported in previous ORFRC sediment diversity studies, members of this family have been detected in other terrestrial and freshwater environments but their ecological function remains unknown [72], [73].

Within environments such as the ORFRC that are defined by acidic-to-circumneutral subsurface regions, thermodynamic modeling of PO_4_^3−^ species in ORFRC groundwater containing two different concentrations of P (e.g., 500 µM and 5 mM) demonstrates the formation of hydroxyapatite across a wide pH range (Figure S2A and S2B), resulting in a secondary path for remediation by providing mineral surface sites for the adsorption of metals and radionuclides. This additional path for phosphate mineral sequestration of U(VI) has been described in a recent study examining microbial hydrolysis of G3P in ORFRC Area 2 synthetic groundwater containing U(VI) and a calcium concentration of 4 mM that resulted in U(VI) coprecipitation with hydroxyapatite [33]. Furthermore, Ca concentrations greater than 1 mM (Figure S2C and S2D) that have been shown to enhance U(VI) transport as well as decrease U(VI) reduction rates [12], [74]–[76]. Thus, harnessing P metabolic capabilities within the ORFRC subsurface that sequester Ca as a mineral phosphate could augment *in situ* U reduction processes. Within this study, the PhyloChip microarray rapidly identified relative abundance changes of prokaryotes with previously characterized P-solubilizing phenotypes as well as several archaeal and bacterial taxa that have yet to be described influences on terrestrial phosphate-cycling. The rapid assessment of microbial community dynamics provided by microarray analyses represents an approach that can provide insight into the diversity of prokaryotes that contribute to terrestrial phosphate-cycling and the influence these taxa could have on the cycling of metals and radionuclides within subsurface environments.

## Materials and Methods

### Ethics Statement

All sediment samples from the U.S. Department of Energy Oak Ridge Field Research Center were requested and obtained from David Watson, Oak Ridge National Laboratory Field Research Manager. This work did not involve field studies nor did it require specific permits.

### Sampling site

Contaminated sediments were collected from the ORFRC (Area 2) located within the Oak Ridge National Laboratory Reservation in Oak Ridge, Tennessee. The contaminated sediments are located adjacent to three former waste ponds (S-3 ponds) used during decades of nuclear weapons production. The ponds and surrounding sediments received uranium, other radionuclides, heavy metals, organic solvents, and nitric acid waste (DOE Subsurface Biogeochemical Research website; http://esd.lbl.gov/research/projects/ersp/). Sediment cores (5 cm internal diameter with an average length of 168 cm) were collected aseptically and preserved under an argon atmosphere. Sediment samples from borehole FB107-04-00 at a depth of 7 meters below ground surface were obtained from the saturated zone where groundwater is approximately 4.5 meters below ground surface (#http://public.ornl.gov/orifc/sitenarrative.cfm#Anchor12). Sediments from borehole FB107-04-00 were used for all incubations. Sediment from 7 meters below ground surface was aseptically subsampled, placed in a sterile plastic bag and homogenized. All subsequent analyses and slurry treatments utilized subsampled homogenized FB107-04-00 sediments. Sediment porewater was pH 6.8, measured with an Orion Dual Star digital meter and calibrated electrode (Thermo Scientific, Beverly, MA). Prior to treatments, carbon (C) content was measured before and after acidification with a Leco CNS 2000 analyzer (Leco Corporation, St. Joseph, MI) at the University of Georgia College of Agricultural and Environmental Services Laboratories. Total C, organic C, and inorganic C content was 1077, 83, and 993 ppm, respectively.

### Sediment slurry incubations and DNA extractions

Sediment slurry treatments were conducted in acid washed 1 L glass Erlenmeyer flasks containing 4 g sediment and synthetic groundwater in a final volume of 250 mL. Synthetic groundwater consisted of: 2 µM FeSO_4_, 5 µM MnCl_2_, 8 µM Na_2_MoO_4_, 0.8 mM MgSO_4_, 7.5 mM NaNO_3_, 0.4 mM KCl, 7.5 mM KNO_3_, and 0.2 mM Ca(NO_3_)_2_. Sediment slurry pH 5.5 treatments were buffered with 50 mM 2-(N-Morpholino) ethanesulfonic acid (Sigma Aldrich, St. Louis, MO) and pH 6.8 treatments were unbuffered. Either G2P or G3P (Sigma Aldrich, St. Louis, MO) were added to sediment slurries as the sole C and P amendment at a final concentration of 10 mM. Control sediment slurry treatments were conducted at pH 5.5 and 6.8 without organophosphate additions. The combinations of pH and organophosphate amendments yielded six different treatment conditions: (1) unamended control (pH 5.5), (2) G2P (pH 5.5), (3) G3P (pH 5.5), (4) unamended control (pH 6.8), (5) G2P (pH 6.8), and (6) G3P (pH 6.8). To maintain oxic growth conditions, sediment slurries were constantly mixed in the dark with a magnetic stir bar at 200 rpm on a Variomag Multipoint 15 magnetic stirrer (Thermo Scientific, Beverly, MA) at 22°C. Aseptic techniques were followed during assembly and sub-sampling of all treatments. All sediment slurry treatments were conducted in triplicate and all subsequent analyses utilized all replicates from each respective treatment. Once incubations were completed, all replicate sediment slurries were centrifuged at 10,000 g for 10 min, supernatant decanted. MP Biomedicals FastDNA spin kit for soils (MP Biomedicals, Solon, OH) was utilized according to manufacturer's protocol to extract genomic DNA from 500 mg of homogenized sediment prior to treatment (subsampled in triplicate) as well as pelleted sediment from each replicate sediment slurry treatment. DNA concentrations for each replicate DNA extraction were measured via absorption at 260 nm using a NanoDrop ND-1000 (Thermo Scientific, Beverly, MA).

### PCR amplification of 16S rRNA genes and PhyloChip analysis

Genomic DNA (gDNA) extracted from each replicate sediment slurry treatment was utilized as template for 16S rRNA gene polymerase chain reaction (PCR). Universal bacterial (27F and 1492R) and universal archaeal (A340F and A934R) primers were utilized for PCR amplification of 16S rRNA of gDNA extractions from all replicate treatments [77]–[79]. Reagents for all PCR reactions and thermocycling conditions for bacterial 16S rRNA genes were performed as previously described [55]. Archaeal PCR conditions consisted of an initial denaturation at 95°C (5 min), 35 cycles of 95°C (30 sec), 60°C (2 min), 72°C (2 min), and a final extension at 72°C (10 min). Purification of replicate16S rRNA gene amplicons were performed as previously described [55].

Archaeal and bacterial 16S rRNA gene diversity present within each of the replicate sediment slurry treatments was assessed using the Affymetrix PhyloChip microarray (i.e., a total of 21 archaeal and 21 bacterial 16S rRNA PCR amplicons obtained from replicate sediment slurry treatment as well as each replicate sediment sample prior to treatment were analyzed on 42 separate microarrays). Microarray sample preparation, hybridization, and normalization were performed as previously described [41], [55]. The threshold for identifying an operational taxonomic unit (OTU) present in a sample was a positive fraction (pf) ≥0.9, indicating that over 90% of perfect match probes from the entire probe set of a given OTU were positive. Total richness each for each sample was determined by summation of all OTUs with a pf ≥0.9. The fold change in community richness of a treatment at a given pH was determined by dividing the average richness of the replicate unamended control treatments by the average richness of the replicate amended treatments. Student t-test was performed and p-value of ≤0.05 was used as cutoff for OTUs with significantly increasing or decreasing abundance based on treatment.

Student t-tests (R Development Core Team, 2011 and PASW Statistics 18 for Microsoft Windows) were performed to determine significance of treatments on OTU fluorescence and community richness. Bray-Curtis distance, non-metric multidimensional scaling (NMDS), and multiple response permutation procedure (MRPP) calculations were performed using the R software platform. NMDS and MRPP groups were defined by treatment (i.e., pH and organophosphate substrate), and both utilized Bray-Curtis distance matrices (1000 permutations). MRPP analysis was performed to test differences among the archaeal and bacterial communities based on treatments. Significance of community differences were calculated from weighted mean within-group observed distances (δ_o_) and expected distances (δ_e_). Chance-corrected within-group agreement (A) was also conducted to assess group similarities where the maximum value of A = 1 indicates all members within groups are identical, A>0 indicates homogeneity, and A<0 indicates heterogeneity [80]. Venn diagrams were constructed using Venny software [81].

### Nutrients, metal and radionuclide measurements

Nutrient measurements (nitrate, nitrite, phosphate, G2P, and G3P) were measured with an ICS-2000 ion chromatography system with an AS-DV automated sampler (Dionex, Sunnyvale, CA) equipped with a degasser, a KOH eluent generator with a continuously regenerating anion trap column, AS11-HC (4×250 mm) anion exchange column, AG11-HC guard column (4×250 mm), ARS 300 4 mm anion regenerating suppressor (164 mA current setting), and Chromeleon 6.8 software. A 25 µl sample loop was used for all samples. The KOH eluent was delivered at a flow rate of 1.25 mL min^−1^ as follows: 0–4 min isocratic (10 mM); 5–20 min gradient (10 mM to 45 mM); 20–23 min isocratic (45 mM); 23–24 min gradient (45 mM to 10 mM). The samples were filtered through a 0.2 µm polyethersulfone membrane (Millipore, Billerica, MA) before analyses. Prior to sediment slurry incubations, nitrate was extracted from 2 g sediment with 2 mL water (18.2 MΩ) by constant agitation with a cell mixer (New Brunswick Scientific, Edison, NJ) for 1 h at room temperature. Nitrate concentration was 39 mg kg^−1^ and nitrite was not detected.

Total dissolved uranium was measured by inductively-coupled plasma mass spectrometry (ICP-MS) with an Agilent 7500a Series system. Blanks, calibration check standards (95–105% recovery), and River Water Certified Reference Material for Trace Metals (SLRS-4, National Research Council Canada, Ottawa, Canada) were analyzed for quality controls. The analytical error on triplicate samples was <3% relative standard deviation (RSD). Sediments (2 g) were digested in 10 mL of 2% nitric acid (trace metal grade, Fisher Scientific, Pittsburgh, PA) for 1 h at room temperature under constant agitation with a cell mixer (New Brunswick Scientific, Edison, NJ). Samples were filtered through a 0.2 µm polyethersulfone membrane (Millipore, Billerica, MA) and diluted in 18.2 MΩ water (Nanopure; Barnstead International, Dubuque, IA). Sediment uranium concentration was 19 mg kg^−1^.

### Thermodynamic modeling

Thermodynamic equilibrium modeling of ORFRC groundwater was conducted using MINEQL+ v. 4.5 [82] updated with the Nuclear Energy Agency's thermodynamic database for uranium [83]. The equilibrium model was developed using the average concentrations of dissolved ions from GW-836 including calcium (4.5 mM), uranium (4.21 µM), carbonate (5 mM), and phosphate (500 µM and 5 mM). GW-836 is the closest groundwater monitoring well proximal to borehole FB107-04-00 (http://public.ornl.gov/orifc/history.cfm?Location='GW-836').

## Supporting Information

Figure S1 **Venn diagram of OTUs enriched in multiple treatments.** (A) Archaeal and (B) bacterial OTUs detected in one or more of the organophosphate-amended treatments. Only OTUs that had a 2-fold or greater increase in fluorescence for each respective treatment were used for comparisons.
(PPTX)

Figure S2 **Thermodynamic modeling of P and Ca in the absence of U(VI) as a function of pH.** ORFRC Area 2 groundwater concentrations of dissolved ions (GW-836 monitoring well), U(VI) = 4.5 µM, and Ca^2+^ = 4.85 mM were used to model the distribution of PO_4_^3−^ species with (A) PO_4_^3−^ = 500 µM, (B) PO_4_^3−^ = 5 mM as well as the distribution of Ca^2+^ species with (C) PO_4_^3−^ = 500 µM and (D) PO_4_^3−^ = 5 mM. Dashed lines represent soluble species and solid lines represent insoluble species.
(PPTX)

Table S1 **Total archaeal and bacterial OTUs detected in ORFRC sediments prior to treatment and following each of the six treatment conditions.** Positive fraction and normalized fluorescence values are reported for each of the triplicate treatments.
(XLS)

Table S2 **OTUs with a 2-fold or greater relative increase in fluorescence intensity following sediment slurry treatments.**
(DOC)

Table S3 **OTUs with a 2-fold or greater relative increase in fluorescence intensity detected in two or more treatments.**
(DOC)
