# Supplementary figures and images for: Microbial Community Responses to Organophosphate Substrate Additions in Contaminated Subsurface Sediments
Source: PLoS One. 2014 Jun 20;9(6):e100383. doi: 10.1371/journal.pone.0100383 (PMC4065101; doi:10.1371/journal.pone.0100383)

## Slide 1
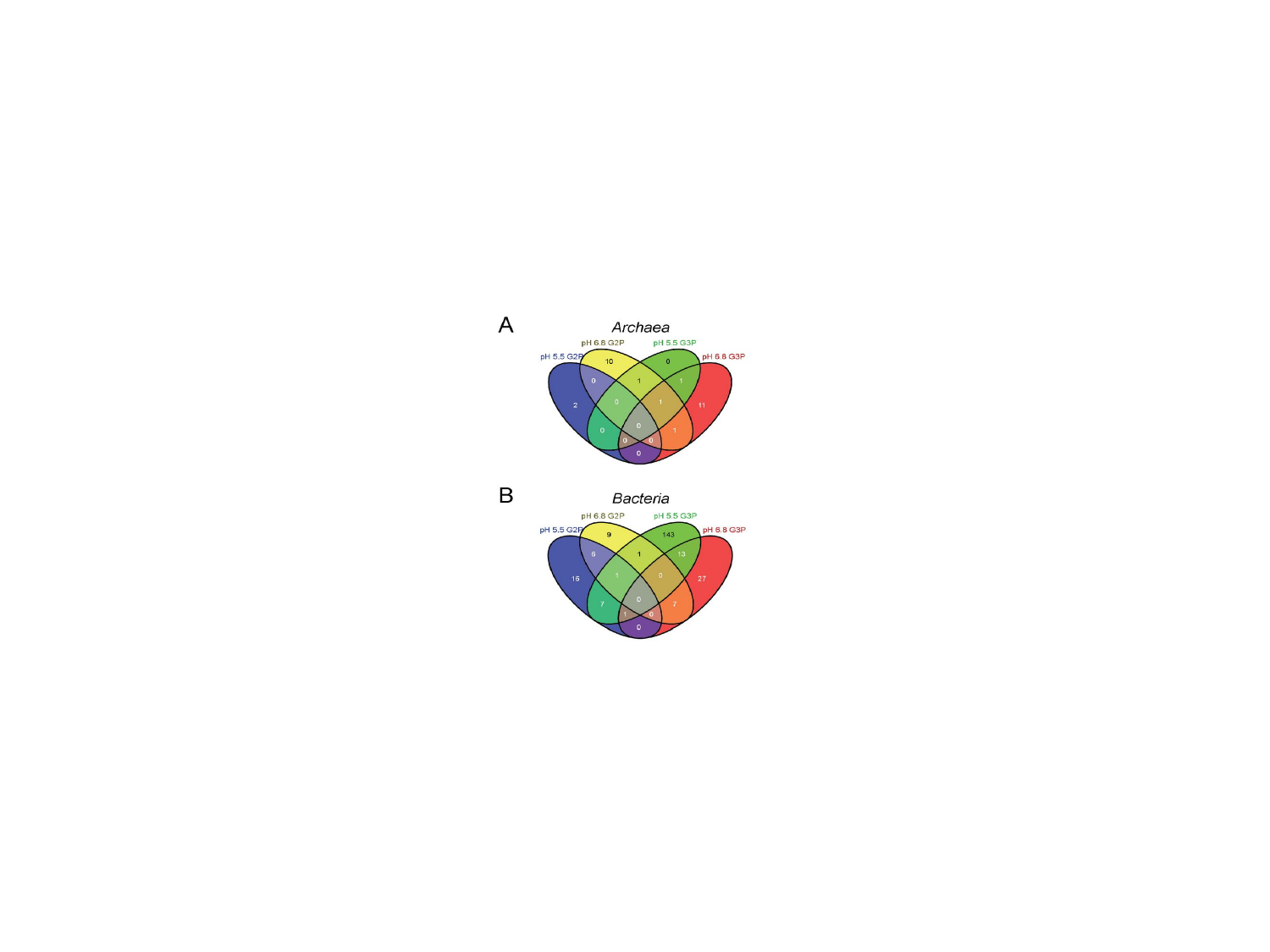

Supplement: Figure S1 — Venn diagram of OTUs enriched in multiple treatments. (A) Archaeal and (B) bacterial OTUs detected in one or more of the organophosphate-amended treatments. Only OTUs that had a 2-fold or greater increase in fluorescence for each respective treatment were used for comparisons. (PPTX) [file pone.0100383.s001.pptx]

## Slide 1
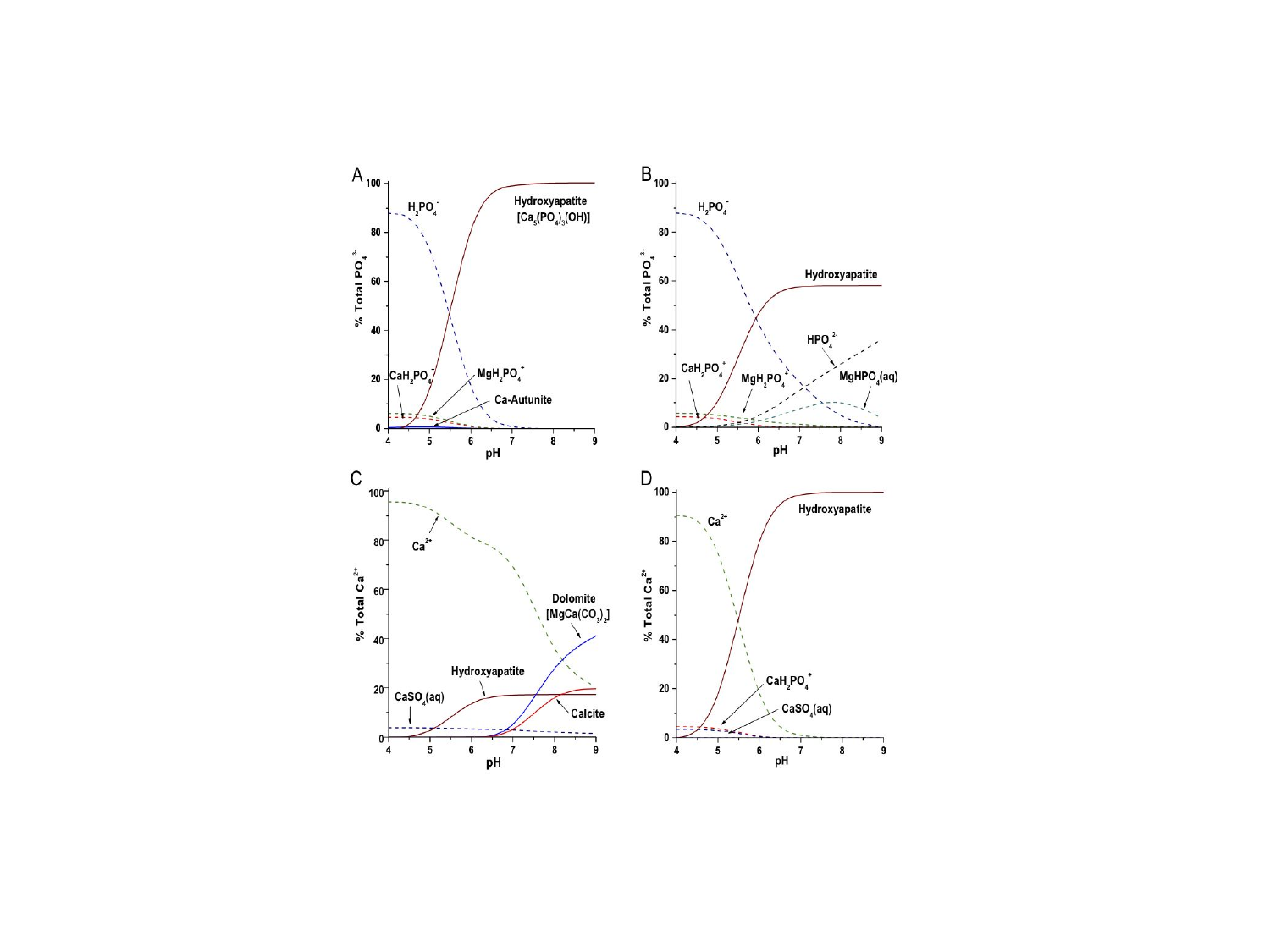

Supplement: Figure S2 — Thermodynamic modeling of P and Ca in the absence of U(VI) as a function of pH. ORFRC Area 2 groundwater concentrations of dissolved ions (GW-836 monitoring well), U(VI) = 4.5 µM, and Ca2+ = 4.85 mM were used to model the distribution of PO4 3− species with (A) PO4 3− = 500 µM, (B) PO4 3− = 5 mM as well as the distribution of Ca2+ species with (C) PO4 3− = 500 µM and (D) PO4 3− = 5 mM. Dashed lines represent soluble species and solid lines represent insoluble species. (PPTX) [file pone.0100383.s002.pptx]
