# Supplementary material for: Microbial Community Responses to Organophosphate Substrate Additions in Contaminated Subsurface Sediments
Source: PLoS One. 2014 Jun 20;9(6):e100383. doi: 10.1371/journal.pone.0100383 (PMC4065101; doi:10.1371/journal.pone.0100383)
Supplement: Table S2 — OTUs with a 2-fold or greater relative increase in fluorescence intensity following sediment slurry treatments. (DOC) [file pone.0100383.s004.doc]

**Supplemental Table S2.** OTUs with a 2-fold or greater relative increase in fluorescence intensity following soil slurry treatments.

| Treatment | Phylum | Class | Order | Family | PhyloChip ID^a^ | GenBank Accession | Fold Increase |
| --- | --- | --- | --- | --- | --- | --- | --- |
| G2P (pH 5.5) | *Crenarchaeota* | Unclassified | Unclassified | Unclassified | sfA 9548^b^ | AY505052.1 | 2.1 |
|  |  | Unclassified | Unclassified | Unclassified | sfA 9156 | AB193986.1 | 2.0 |
|  | *Nitrospira* | *Nitrospira* | *Nitrospirales* | *Nitrospiraceae* | sf_1 864 | Y14644.1 | 3.0 |
|  | *Proteobacteria* | *α-proteobacteria* | *Caulobacterales* | *Caulobacteraceae* | sf_1 7578^b^ | AJ227774.1 | 7.0 |
|  |  | *α-proteobacteria* | *Caulobacterales* | *Caulobacteraceae* | sf_1 7134^b^ | AF236003.1 | 2.8 |
|  |  | *α-proteobacteria* | *Caulobacterales* | *Caulobacteraceae* | sf_1 6929^b^ | Y18216.1 | 2.7 |
|  |  | *α-proteobacteria* | *Caulobacterales* | *Caulobacteraceae* | sf_1 6953 | AJ227767.1 | 2.0 |
|  |  | *α-proteobacteria* | *Rhizobiales* | *Hyphomicrobiaceae* | sf_1 7298^b^ |  | 16.9 |
|  |  | *α-proteobacteria* | *Rhizobiales* | *Phyllobacteriaceae* | sf_1 6854^b^ |  | 7.1 |
|  |  | *α-proteobacteria* | *Rhizobiales* | *Phyllobacteriaceae* | sf_1 6876^b^ | AF003376.1 | 4.7 |
|  |  | *α-proteobacteria* | *Rhizobiales* | *Phyllobacteriaceae* | sf_1 6962 | AJ132378.1 | 3.2 |
|  |  | *α-proteobacteria* | *Rhizobiales* | *Phyllobacteriaceae* | sf_1 7543 | AJ318199.1 | 2.2 |
|  |  | *α-proteobacteria* | *Rhizobiales* | *Rhizobiaceae* | sf_1 6725^b^ | AL591782.1 | 3.0 |
|  |  | *α-proteobacteria* | *Rhizobiales* | *Rhizobiaceae* | sf_1 7380^b^ | AB118158.1 | 2.4 |
|  |  | *α-proteobacteria* | *Rhizobiales* | *Rhizobiaceae* | sf_1 6847^b^ | U29387.1 | 6.1 |
|  |  | *α-proteobacteria* | *Rhizobiales* | Unclassified | sf_1 7255 | AB159685.1 | 3.5 |
|  |  | *α-proteobacteria* | *Rhodobacterales* | *Rhodobacteraceae* | sf_1 6979^b^ | AF234741.1 | 2.2 |
|  |  | *α-proteobacteria* | *Rhodospirillales* | *Rhodospirillaceae* | sf_1 7109^b^ | AY189753.1 | 2.1 |
|  |  | *α-proteobacteria* | *Sphingomonadales* | *Sphingomonadaceae* | sf_1 7183^b^ | AF468353.1 | 2.4 |
|  |  | *α-proteobacteria* | Unclassified | Unclassified | sf_3 7010 | AJ428412.1 | 2.2 |
|  |  | *β-proteobacteria* | *Burkholderiales* | *Comamonadaceae* | sf_1 7834^b^ | AB021418.1 | 4.3 |
|  |  | *β-proteobacteria* | *Burkholderiales* | *Comamonadaceae* | sf_1 7882 | AF526937.1 | 3.4 |
|  |  | *β-proteobacteria* | *Rhodocyclales* | *Rhodocyclaceae* | sf_1 7980 | AF204243.1 | 2.1 |
|  |  | *δ-proteobacteria* | *Desulfobacterales* | *Desulfobacteraceae* | sf_5 9821 |  | 4.4 |
|  |  | *δ-proteobacteria* | *Myxococcales* | *Polyangiaceae* | sf_3 9755 | AF382126.1 | 2.2 |
|  |  | *γ-proteobacteria* | *Chromatiales* | *Ectothiorhodospiraceae* | sf_1 9451^b^ | X93480.1 | 2.5 |
|  |  | *γ-proteobacteria* | *Enterobacteriales* | *Enterobacteriaceae* | sf_1 8758^b^ | U80201.1 | 4.6 |
|  |  | *γ-proteobacteria* | *Pseudomonadales* | *Pseudomonadaceae* | sf_1 8508 | AF530073.1 | 2.0 |
|  | *Proteobacteria* | *γ-proteobacteria* | *Thiotrichales* | *Piscirickettsiaceae* | sf_3 8845^b^ | AF513949.1 | 2.4 |
|  |  | *γ-proteobacteria* | *Xanthomonadales* | *Xanthomonadaceae* | sf_3 9150 |  | 3.0 |
|  |  | *γ-proteobacteria* | *Xanthomonadales* | *Xanthomonadaceae* | sf_3 9093^b^ |  | 2.3 |
|  |  | *γ-proteobacteria* | *Xanthomonadales* | *Xanthomonadaceae* | sf_3 8538^b^ |  | 2.0 |

**Supplemental Table S2.** Cont.

| Treatment | Phylum | Class | Order | Family | PhyloChip ID^a^ | GenBank Accession | Fold Increase |
| --- | --- | --- | --- | --- | --- | --- | --- |
|  | Unclassified | Unclassified | Unclassified | Unclassified | sf_160 7127^b^ |  | 2.7 |
| G2P (pH 6.8) | *Crenarchaeota* | Unclassified | Unclassified | Unclassified | sfA 9126 | AJ576215.1 | 2.4 |
|  |  | Unclassified | Unclassified | Unclassified | sfA 9131 | AY592502.1 | 3.8 |
|  | *Euryarchaeota* | *Methanobacteria* | *Methanobacteriales* | *Methanobacteriaceae* | sfA 9680 | AY351489.1 | 5.5 |
|  |  | *Methanobacteria* | *Methanobacteriales* | *Methanobacteriaceae* | sfA 9302 | U82322.1 | 5.5 |
|  |  | *Methanobacteria* | *Methanobacteriales* | *Methanobacteriaceae* | sfA 9150 | AY552778.3 | 3.8 |
|  |  | *Methanobacteria* | *Methanobacteriales* | *Methanobacteriaceae* | sfA 9641 | AY351445.1 | 2.4 |
|  |  | *Methanobacteria* | *Methanobacteriales* | *Methanobacteriaceae* | sfA 9370 | AY351478.1 | 2.4 |
|  |  | *Methanobacteria* | *Methanobacteriales* | *Methanobacteriaceae* | sfA 9912 | AY351446.1 | 2.4 |
|  |  | *Methanobacteria* | *Methanobacteriales* | *Methanobacteriaceae* | sfA 9075 | AF242652.1 | 2.4 |
|  |  | *Methanobacteria* | *Methanobacteriales* | *Methanobacteriaceae* | sfA 10138 | AY351444.1 | 2.4 |
|  |  | *Methanobacteria* | *Methanobacteriales* | *Methanobacteriaceae* | sfA 9407 | AY351484.1 | 2.4 |
|  |  | *Methanobacteria* | *Methanobacteriales* | MSBL1 | sfC 9702 | AY627495.1 | 2.6 |
|  | Unclassified | Unclassified | Unclassified | Unclassified | sfA 9377 | AF356635.1 | 2.3 |
|  | *Acidobacteria* | *Holophagae* | *Holophagales* | *Holophagaceae* | sf_14 208 | AJ519665.1 | 2.1 |
|  |  | *Chloracidobacteria* | Unclassified | Unclassified | sf_1 790 | Z95709.1 | 2.0 |
|  | *Bacteroidetes* | *Bacteroidetes* | *Bacteroidales* | *Prevotellaceae* | sf_1 6152^b^ | AF001768.1 | 5.9 |
|  |  | *Bacteroidetes* | *Bacteroidales* | *Prevotellaceae* | sf_1 6236^b^ |  | 4.7 |
|  |  | *Bacteroidetes* | *Bacteroidales* | Unclassified | sf_15 6069 | AJ289174.1 | 2.8 |
|  |  | *Sphingobacteria* | *Sphingobacteriales* | Unclassified | sf_11 5491^b^ | AJ290025.1 | 3.1 |
|  |  | *Sphingobacteria* | *Sphingobacteriales* | Unclassified | sf_11 5987^b^ |  | 2.5 |
|  |  | *Sphingobacteria* | *Sphingobacteriales* | Unclassified | sf_11 6050 | AF527580.1 | 2.1 |
|  |  | *Cytophagia* | *Cytophagales* | Unclassified | sf_19 6055^b^ | AF507701.1 | 4.4 |
|  |  | *Cytophagia* | *Cytophagales* | Unclassified | sf_19 5368^b^ | AB078071.1 | 2.7 |
|  | *Chloroflexi* | *Chloroflexi* | *Roseiflexales* | Unclassified | sf_5 1048 | X84565.2 | 2.0 |
|  | *Deferribacteres* | *Deferribacteres* | Unclassified | Unclassified | sf_1 797 | AJ515881.1 | 2.1 |
|  | *Nitrospira* | *Nitrospira* | *Nitrospirales* | *Nitrospiraceae* | sf_1 864 | Y14644.1 | 2.5 |
|  | *Planctomycetes* | *Planctomycetacia* | *Planctomycetales* | Unclassified | sf_4 9662 | AF407728.1 | 2.3 |
|  | *Proteobacteria* | *α-proteobacteria* | *Rhizobiales* | *Phyllobacteriaceae* | sf_1 6876^b^ | AF003376.1 | 2.3 |
|  |  | *α-proteobacteria* | *Rhizobiales* | *Phyllobacteriaceae* | sf_1 6962 | AJ132378.1 | 2.2 |
|  |  | *α-proteobacteria* | *Rhizobiales* | *Phyllobacteriaceae* | sf_1 6854^b^ |  | 2.0 |
|  |  | *α-proteobacteria* | *Rhizobiales* | *Rhizobiaceae* | sf_1 6847^b^ | U29387.1 | 2.8 |
|  |  | *α-proteobacteria* | *Rhizobiales* | *Rhizobiaceae* | sf_1 7380^b^ | AB118158.1 | 2.8 |

**Supplemental Table S2.** Cont.

| Treatment | Phylum | Class | Order | Family | PhyloChip ID^a^ | GenBank Accession | Fold Increase |
| --- | --- | --- | --- | --- | --- | --- | --- |
|  |  | *α-proteobacteria* | *Rhizobiales* | *Rhizobiaceae* | sf_1 6729^b^ | AF441727.1 | 2.8 |
|  |  | *α-proteobacteria* | *Rhizobiales* | *Rhizobiaceae* | sf_1 6725^b^ | AL591782.1 | 2.7 |
|  |  | *α-proteobacteria* | *Sphingomonadales* | *Sphingomonadaceae* | sf_1 7404^b^ | AF410927.1 | 2.1 |
|  |  | *γ-proteobacteria* | *Enterobacteriales* | *Enterobacteriaceae* | sf_6 103^b^ |  | 15.5 |
|  | Unclassified | Unclassified | Unclassified | Unclassified | sf_160 539^b^ |  | 3.3 |
| G3P (pH5.5) | *Crenarchaeota* | Unclassified | Unclassified | Unclassified | sfA 9131 | AY592502.1 | 2.1 |
|  | *Euryarchaeota* | *Archaeoglobi* | *Archaeoglobales* | *Archaeoglobaceae* | sfA 9182 | AB019735.1 | 2.4 |
|  |  | *Methanobacteria* | *Methanobacteriales* | MSBL1 | sfC 9702 | AY627495.1 | 2.6 |
|  | *Deferribacteres* | *Deferribacer* | Unclassified | Unclassified | sf_1 797 | AJ515881.1 | 2.1 |
|  | *Firmicutes* | *Bacilli* | *Bacillales* | *Bacillaceae* | sf_1 3900 | X68416.1 | 3.2 |
|  | *Proteobacteria* | *α-proteobacteria* | *Rhizobiales* | *Methylocystaceae* | sf_1 7255 | AB159685.1 | 2.7 |
|  |  | *α-proteobacteria* | *Rhizobiales* | *Phyllobacteriaceae* | sf_1 6854^b^ |  | 4.5 |
|  |  | *α-proteobacteria* | *Rhizobiales* | Unclassified | sf_3 7401^b^ | AF359545.1 | 2.3 |
|  |  | *α-proteobacteria* | *Rhodobacterales* | *Rhodobacteraceae* | sf_1 7536^b^ |  | 3.9 |
|  |  | *α-proteobacteria* | *Rhodobacterales* | *Rhodobacteraceae* | sf_1 6991 | X53853.1 | 2.0 |
|  |  | *α-proteobacteria* | *Rhodospirillales* | *Rhodospirillaceae* | sf_3 7024^b^ | AY186195.1 | 2.0 |
|  |  | *α-proteobacteria* | *Rhodospirillales* | *Rhodospirillaceae* | sf_1 7109^b^ | AY189753.1 | 3.3 |
|  |  | *β-proteobacteria* | *Burkholderiales* | *Comamonadaceae* | sf_1 7882 | AF526937.1 | 3.9 |
|  |  | *β-proteobacteria* | *Burkholderiales* | *Comamonadaceae* | sf_1 7834^b^ | AB021418.1 | 3.7 |
|  |  | *β-proteobacteria* | *Burkholderiales* | *Comamonadaceae* | sf_1 8025 | AJ292624.1 | 2.1 |
|  |  | *β-proteobacteria* | *Hydrogenophilales* | *Hydrogenophilaceae* | sf_2 8756^b^ | X97534.1 | 2.7 |
|  |  | *γ-proteobacteria* | *Aeromonadales* | *Aeromonadaceae* | sf_1 9494^b^ | AY532691.1 | 2.5 |
|  |  | *γ-proteobacteria* | *Aeromonadales* | *Aeromonadaceae* | sf_1 9000^b^ | AF170914.4 | 2.3 |
|  |  | *γ-proteobacteria* | *Aeromonadales* | *Aeromonadaceae* | sf_1 8621 | AF427150.1 | 2.1 |
|  |  | *γ-proteobacteria* | *Aeromonadales* | *Aeromonadaceae* | sf_1 9026^b^ | AJ009860.1 | 2.1 |
|  |  | *γ-proteobacteria* | *Aeromonadales* | *Aeromonadaceae* | sf_1 8364^b^ | S39232.1 | 2.0 |
|  |  | *γ-proteobacteria* | *Aeromonadales* | *Aeromonadaceae* | sf_1 8340^b^ | X71120.1 | 2.0 |
|  |  | *γ-proteobacteria* | *Aeromonadales* | *Aeromonadaceae* | sf_1 9440^b^ | X60412.1 | 2.0 |
|  |  | *γ-proteobacteria* | *Alteromonadales* | *Alteromonadaceae* | sf_1 9247^b^ | AF500078.1 | 4.3 |
|  |  | *γ-proteobacteria* | *Alteromonadales* | *Alteromonadaceae* | sf_1 9227^b^ |  | 3.9 |
|  |  | *γ-proteobacteria* | *Alteromonadales* | *Alteromonadaceae* | sf_1 9586^b^ | AY190533.1 | 2.8 |
|  |  | *γ-proteobacteria* | *Alteromonadales* | *Alteromonadaceae* | sf_1 9288 | AJ295715.1 | 2.7 |

**Supplemental Table S2.** Cont.

| Treatment | Phylum | Class | Order | Family | PhyloChip ID^a^ | GenBank Accession | Fold Increase |
| --- | --- | --- | --- | --- | --- | --- | --- |
|  |  | *γ-proteobacteria* | *Alteromonadales* | *Alteromonadaceae* | sf_1 9222^b^ | X82132.1 | 2.6 |
|  |  | *γ-proteobacteria* | *Alteromonadales* | *Alteromonadaceae* | sf_1 9218 | X67024.1 | 2.5 |
|  |  | *γ-proteobacteria* | *Alteromonadales* | *Alteromonadaceae* | sf_1 8222 |  | 2.5 |
|  |  | *γ-proteobacteria* | *Alteromonadales* | *Alteromonadaceae* | sf_1 8643 | AY771715.1 | 2.4 |
|  |  | *γ-proteobacteria* | *Alteromonadales* | *Alteromonadaceae* | sf_1 8975 | AB016268.1 | 2.4 |
|  |  | *γ-proteobacteria* | *Alteromonadales* | *Alteromonadaceae* | sf_1 9143 | AJ417594.1 | 2.3 |
|  |  | *γ-proteobacteria* | *Alteromonadales* | *Alteromonadaceae* | sf_1 8174^b^ | AF114499.1 | 2.3 |
|  |  | *γ-proteobacteria* | *Alteromonadales* | *Alteromonadaceae* | sf_1 9067 | AF006669.1 | 2.3 |
|  |  | *γ-proteobacteria* | *Alteromonadales* | *Alteromonadaceae* | sf_1 9640 | AY028205.1 | 2.3 |
|  |  | *γ-proteobacteria* | *Alteromonadales* | *Alteromonadaceae* | sf_1 8732^b^ | AY205304.1 | 2.3 |
|  |  | *γ-proteobacteria* | *Alteromonadales* | *Alteromonadaceae* | sf_1 9411 | AY170366.1 | 2.2 |
|  |  | *γ-proteobacteria* | *Alteromonadales* | *Alteromonadaceae* | sf_1 8580 | AJ295713.1 | 2.2 |
|  |  | *γ-proteobacteria* | *Alteromonadales* | *Alteromonadaceae* | sf_1 8579 | AJ416756.1 | 2.2 |
|  |  | *γ-proteobacteria* | *Alteromonadales* | *Alteromonadaceae* | sf_1 9058 | X82136.1 | 2.2 |
|  |  | *γ-proteobacteria* | *Alteromonadales* | *Alteromonadaceae* | sf_1 8932 | AF045560.1 | 2.2 |
|  |  | *γ-proteobacteria* | *Alteromonadales* | *Alteromonadaceae* | sf_1 8916 | U91545.1 | 2.2 |
|  |  | *γ-proteobacteria* | *Alteromonadales* | *Alteromonadaceae* | sf_1 9384 | AJ132226.1 | 2.1 |
|  |  | *γ-proteobacteria* | *Alteromonadales* | *Alteromonadaceae* | sf_1 8336 | AF237977.1 | 2.1 |
|  |  | *γ-proteobacteria* | *Alteromonadales* | *Alteromonadaceae* | sf_1 9386 | AB016267.1 | 2.1 |
|  |  | *γ-proteobacteria* | *Alteromonadales* | *Alteromonadaceae* | sf_1 8904^b^ | AB059263.1 | 2.1 |
|  |  | *γ-proteobacteria* | *Alteromonadales* | *Alteromonadaceae* | sf_1 8753 | AY553079.1 | 2.1 |
|  |  | *γ-proteobacteria* | *Alteromonadales* | *Shewanellaceae* | sf_1 8581^b^ | AB008796.1 | 2.6 |
|  |  | *γ-proteobacteria* | *Alteromonadales* | *Shewanellaceae* | sf_1 8641 | AJ252022.1 | 2.0 |
|  |  | *γ-proteobacteria* | *Alteromonadales* | *Shewanellaceae* | sf_1 8662 |  | 3.0 |
|  |  | *γ-proteobacteria* | *Alteromonadales* | *Shewanellaceae* | sf_1 9081 | AB059264.1 | 2.9 |
|  |  | *γ-proteobacteria* | *Alteromonadales* | *Shewanellaceae* | sf_1 9344 | AF005249.1 | 2.1 |
|  |  | *γ-proteobacteria* | *Chromatiales* | *Chromatiaceae* | sf_1 9370 | AB010860.1 | 2.8 |
|  |  | *γ-proteobacteria* | *Enterobacteriales* | *Enterobacteriaceae* | sf_1 9082^b^ | AJ223469.1 | 405.7 |
|  |  | *γ-proteobacteria* | *Enterobacteriales* | *Enterobacteriaceae* | sf_1 9276^b^ | AJ233422.1 | 215.3 |
|  |  | *γ-proteobacteria* | *Enterobacteriales* | *Enterobacteriaceae* | sf_1 8173^b^ | Z76752.1 | 39.5 |
|  |  | *γ-proteobacteria* | *Enterobacteriales* | *Enterobacteriaceae* | sf_1 8564 | X79939.1 | 24.1 |
|  |  | *γ-proteobacteria* | *Enterobacteriales* | *Enterobacteriaceae* | sf_1 8758^b^ | U80201.1 | 18.5 |

**Supplemental Table S2.** Cont.

| Treatment | Phylum | Class | Order | Family | PhyloChip ID^a^ | GenBank accession | Fold Increase |
| --- | --- | --- | --- | --- | --- | --- | --- |
|  |  | *γ-proteobacteria* | *Enterobacteriales* | *Enterobacteriaceae* | sf_1 1206^b^ | AY265343.1 | 12.5 |
|  |  | *γ-proteobacteria* | *Enterobacteriales* | *Enterobacteriaceae* | sf_1 8640^b^ |  | 7.0 |
|  |  | *γ-proteobacteria* | *Enterobacteriales* | *Enterobacteriaceae* | sf_1 9420^b^ |  | 7.0 |
|  |  | *γ-proteobacteria* | *Enterobacteriales* | *Enterobacteriaceae* | sf_1 8885^b^ | AB089246.1 | 5.9 |
|  |  | *γ-proteobacteria* | *Enterobacteriales* | *Enterobacteriaceae* | sf_1 9651^b^ | AF373176.1 | 5.6 |
|  |  | *γ-proteobacteria* | *Enterobacteriales* | *Enterobacteriaceae* | sf_1 8716^b^ | AB033602.1 | 5.6 |
|  |  | *γ-proteobacteria* | *Enterobacteriales* | *Enterobacteriaceae* | sf_1 8473^b^ | M59155.1 | 5.5 |
|  |  | *γ-proteobacteria* | *Enterobacteriales* | *Enterobacteriaceae* | sf_1 8528^b^ | Y17665.1 | 5.4 |
|  |  | *γ-proteobacteria* | *Enterobacteriales* | *Enterobacteriaceae* | sf_1 8590^b^ | AJ010486.1 | 5.1 |
|  |  | *γ-proteobacteria* | *Enterobacteriales* | *Enterobacteriaceae* | sf_1 9293^b^ | AJ245598.1 | 5.0 |
|  |  | *γ-proteobacteria* | *Enterobacteriales* | *Enterobacteriaceae* | sf_1 8740^b^ | AF373201.1 | 4.4 |
|  |  | *γ-proteobacteria* | *Enterobacteriales* | *Enterobacteriaceae* | sf_1 8934^b^ | AF373188.1 | 4.4 |
|  |  | *γ-proteobacteria* | *Enterobacteriales* | *Enterobacteriaceae* | sf_1 8530^b^ | AJ489826.1 | 4.2 |
|  |  | *γ-proteobacteria* | *Enterobacteriales* | *Enterobacteriaceae* | sf_1 9265^b^ | AY253919.1 | 4.2 |
|  |  | *γ-proteobacteria* | *Enterobacteriales* | *Enterobacteriaceae* | sf_1 8742^b^ | AF084835.1 | 4.0 |
|  |  | *γ-proteobacteria* | *Enterobacteriales* | *Enterobacteriaceae* | sf_1 8773^b^ |  | 4.0 |
|  |  | *γ-proteobacteria* | *Enterobacteriales* | *Enterobacteriaceae* | sf_6 8783^b^ | AF075271.2 | 3.9 |
|  |  | *γ-proteobacteria* | *Enterobacteriales* | *Enterobacteriaceae* | sf_1 8251^b^ | AF214640.1 | 3.8 |
|  |  | *γ-proteobacteria* | *Enterobacteriales* | *Enterobacteriaceae* | sf_1 9363^b^ | AF025365.1 | 3.8 |
|  |  | *γ-proteobacteria* | *Enterobacteriales* | *Enterobacteriaceae* | sf_1 9274^b^ | AJ550468.1 | 3.7 |
|  |  | *γ-proteobacteria* | *Enterobacteriales* | *Enterobacteriaceae* | sf_1 8362^b^ | AF453251.1 | 3.7 |
|  |  | *γ-proteobacteria* | *Enterobacteriales* | *Enterobacteriaceae* | sf_1 9309 | AJ417833.1 | 3.7 |
|  |  | *γ-proteobacteria* | *Enterobacteriales* | *Enterobacteriaceae* | sf_1 9417^b^ | AJ233429.1 | 3.7 |
|  |  | *γ-proteobacteria* | *Enterobacteriales* | *Enterobacteriaceae* | sf_1 8890^b^ | X93216.1 | 3.7 |
|  |  | *γ-proteobacteria* | *Enterobacteriales* | *Enterobacteriaceae* | sf_1 8510^b^ | AJ233420.1 | 3.6 |
|  |  | *γ-proteobacteria* | *Enterobacteriales* | *Enterobacteriaceae* | sf_1 8505^b^ | AJ233406.1 | 3.6 |
|  |  | *γ-proteobacteria* | *Enterobacteriales* | *Enterobacteriaceae* | sf_1 9302^b^ | AF373198.1 | 3.6 |
|  |  | *γ-proteobacteria* | *Enterobacteriales* | *Enterobacteriaceae* | sf_1 8936^b^ | AF543283.1 | 3.6 |
|  |  | *γ-proteobacteria* | *Enterobacteriales* | *Enterobacteriaceae* | sf_1 9390^b^ | Z96077.1 | 3.5 |
|  |  | *γ-proteobacteria* | *Enterobacteriales* | *Enterobacteriaceae* | sf_1 8711^b^ | AJ233432.1 | 3.5 |
|  |  | *γ-proteobacteria* | *Enterobacteriales* | *Enterobacteriaceae* | sf_1 8700 | AF489427.1 | 3.5 |
|  |  | *γ-proteobacteria* | *Enterobacteriales* | *Enterobacteriaceae* | sf_1 8712^b^ | AJ233434.1 | 3.4 |

**Supplemental Table S2.** Cont.

| Treatment | Phylum | Class | Order | Family | PhyloChip ID^a^ | GenBank Accession | Fold Increase |
| --- | --- | --- | --- | --- | --- | --- | --- |
|  |  | *γ-proteobacteria* | *Enterobacteriales* | *Enterobacteriaceae* | sf_1 9060^b^ | AJ853891.1 | 3.4 |
|  |  | *γ-proteobacteria* | *Enterobacteriales* | *Enterobacteriaceae* | sf_1 8554^b^ | AJ627202.1 | 3.4 |
|  |  | *γ-proteobacteria* | *Enterobacteriales* | *Enterobacteriaceae* | sf_1 9361^b^ | AB004747.1 | 3.4 |
|  |  | *γ-proteobacteria* | *Enterobacteriales* | *Enterobacteriaceae* | sf_1 9345^b^ | AF141891.1 | 3.3 |
|  |  | *γ-proteobacteria* | *Enterobacteriales* | *Enterobacteriaceae* | sf_1 8379^b^ | AF289542.1 | 3.3 |
|  |  | *γ-proteobacteria* | *Enterobacteriales* | *Enterobacteriaceae* | sf_1 8693 | AF130912.1 | 3.2 |
|  |  | *γ-proteobacteria* | *Enterobacteriales* | *Enterobacteriaceae* | sf_1 8529^b^ | AF181574.1 | 3.2 |
|  |  | *γ-proteobacteria* | *Enterobacteriales* | *Enterobacteriaceae* | sf_1 8739^b^ | AJ233427.1 | 3.2 |
|  |  | *γ-proteobacteria* | *Enterobacteriales* | *Enterobacteriaceae* | sf_1 8467^b^ | AB061685.1 | 3.2 |
|  |  | *γ-proteobacteria* | *Enterobacteriales* | *Enterobacteriaceae* | sf_1 9142^b^ | AJ233410.1 | 3.2 |
|  |  | *γ-proteobacteria* | *Enterobacteriales* | *Enterobacteriaceae* | sf_1 9252^b^ | AF130971.2 | 3.1 |
|  |  | *γ-proteobacteria* | *Enterobacteriales* | *Enterobacteriaceae* | sf_1 8892^b^ | U93263.1 | 3.1 |
|  |  | *γ-proteobacteria* | *Enterobacteriales* | *Enterobacteriaceae* | sf_1 8624^b^ | AF476105.1 | 3.1 |
|  |  | *γ-proteobacteria* | *Enterobacteriales* | *Enterobacteriaceae* | sf_1 9496^b^ |  | 3.0 |
|  |  | *γ-proteobacteria* | *Enterobacteriales* | *Enterobacteriaceae* | sf_1 9262^b^ | AJ871363.1 | 2.9 |
|  |  | *γ-proteobacteria* | *Enterobacteriales* | *Enterobacteriaceae* | sf_1 8300^b^ | D78006.1 | 2.8 |
|  |  | *γ-proteobacteria* | *Enterobacteriales* | *Enterobacteriaceae* | sf_1 9358^b^ | U92194.1 | 2.8 |
|  |  | *γ-proteobacteria* | *Enterobacteriales* | *Enterobacteriaceae* | sf_1 8627 | AF476099.1 | 2.8 |
|  |  | *γ-proteobacteria* | *Enterobacteriales* | *Enterobacteriaceae* | sf_1 8886^b^ | AF170176.1 | 2.7 |
|  |  | *γ-proteobacteria* | *Enterobacteriales* | *Enterobacteriaceae* | sf_1 9029^b^ | AY280573.1 | 2.6 |
|  |  | *γ-proteobacteria* | *Enterobacteriales* | *Enterobacteriaceae* | sf_1 8603 | AF476106.1 | 2.6 |
|  |  | *γ-proteobacteria* | *Enterobacteriales* | *Enterobacteriaceae* | sf_1 8642 | AF373202.1 | 2.6 |
|  |  | *γ-proteobacteria* | *Enterobacteriales* | *Enterobacteriaceae* | sf_1 8770^b^ |  | 2.4 |
|  |  | *γ-proteobacteria* | *Enterobacteriales* | *Enterobacteriaceae* | sf_1 8607 | AF476100.1 | 2.4 |
|  |  | *γ-proteobacteria* | *Enterobacteriales* | *Enterobacteriaceae* | sf_1 8282 | AF476101.1 | 2.4 |
|  |  | *γ-proteobacteria* | *Enterobacteriales* | *Enterobacteriaceae* | sf_1 8504 | AF476104.1 | 2.2 |
|  |  | *γ-proteobacteria* | *Enterobacteriales* | *Enterobacteriaceae* | sf_1 9290 | AF465797.1 | 2.0 |
|  |  | *γ-proteobacteria* | *Enterobacteriales* | Unclassified | sf_1 8430^b^ | AF029226.1 | 2.4 |
|  |  | *γ-proteobacteria* | *Oceanospirillales* | *Halomonadaceae* | sf_1 8598 | X92417.1 | 2.4 |
|  |  | *γ-proteobacteria* | *Oceanospirillales* | *Halomonadaceae* | sf_1 8317 | AF211861.1 | 2.2 |
|  |  | *γ-proteobacteria* | *Oceanospirillales* | *Saccharospirillaceae* | sf_1 8889 | AF452603.1 | 3.3 |
|  |  | *γ-proteobacteria* | *Pseudomonadales* | *Pseudomonadaceae* | sf_1 9050^b^ | AF139998.1 | 17.1 |

**Supplemental Table S2.** Cont.

| Treatment | Phylum | Class | Order | Family | PhyloChip ID^a^ | GenBank Accession | Fold Increase |
| --- | --- | --- | --- | --- | --- | --- | --- |
|  |  | *γ-proteobacteria* | *Pseudomonadales* | *Pseudomonadaceae* | sf_1 8635^b^ |  | 9.5 |
|  |  | *γ-proteobacteria* | *Pseudomonadales* | *Pseudomonadaceae* | sf_1 9366 | AJ293826.1 | 4.9 |
|  |  | *γ-proteobacteria* | *Pseudomonadales* | *Pseudomonadaceae* | sf_1 9219 | AF181576.1 | 4.7 |
|  |  | *γ-proteobacteria* | *Pseudomonadales* | *Pseudomonadaceae* | sf_1 8508 | AF530073.1 | 4.3 |
|  |  | *γ-proteobacteria* | *Pseudomonadales* | *Pseudomonadaceae* | sf_1 8725 | AJ419674.1 | 3.0 |
|  |  | *γ-proteobacteria* | *Pseudomonadales* | *Pseudomonadaceae* | sf_1 9238 |  | 2.9 |
|  |  | *γ-proteobacteria* | *Pseudomonadales* | *Pseudomonadaceae* | sf_1 9469 | AF290486.1 | 2.7 |
|  |  | *γ-proteobacteria* | *Pseudomonadales* | *Pseudomonadaceae* | sf_1 8755 | AJ288146.1 | 2.6 |
|  |  | *γ-proteobacteria* | *Pseudomonadales* | *Pseudomonadaceae* | sf_1 9493 | AF430125.1 | 2.6 |
|  |  | *γ-proteobacteria* | *Pseudomonadales* | *Pseudomonadaceae* | sf_1 8691^b^ | AE004501.1 | 2.6 |
|  |  | *γ-proteobacteria* | *Pseudomonadales* | *Pseudomonadaceae* | sf_1 9588^b^ | AF530072.1 | 2.6 |
|  |  | *γ-proteobacteria* | *Pseudomonadales* | *Pseudomonadaceae* | sf_1 9295 |  | 2.5 |
|  |  | *γ-proteobacteria* | *Pseudomonadales* | *Pseudomonadaceae* | sf_1 8344 | AF425998.1 | 2.5 |
|  |  | *γ-proteobacteria* | *Pseudomonadales* | *Pseudomonadaceae* | sf_1 9175 | AF405328.1 | 2.4 |
|  |  | *γ-proteobacteria* | *Pseudomonadales* | *Pseudomonadaceae* | sf_1 8777 | AB095005.1 | 2.4 |
|  |  | *γ-proteobacteria* | *Pseudomonadales* | *Pseudomonadaceae* | sf_1 8433 | AB001441.1 | 2.3 |
|  |  | *γ-proteobacteria* | *Pseudomonadales* | *Pseudomonadaceae* | sf_1 9068 | AF143245.1 | 2.3 |
|  |  | *γ-proteobacteria* | *Pseudomonadales* | *Pseudomonadaceae* | sf_1 8852 | AF063219.1 | 2.3 |
|  |  | *γ-proteobacteria* | *Pseudomonadales* | *Pseudomonadaceae* | sf_1 9028 | AF468452.1 | 2.3 |
|  |  | *γ-proteobacteria* | *Pseudomonadales* | *Pseudomonadaceae* | sf_1 8601 | AB021401.1 | 2.3 |
|  |  | *γ-proteobacteria* | *Pseudomonadales* | *Pseudomonadaceae* | sf_1 8513 | AF064458.1 | 2.3 |
|  |  | *γ-proteobacteria* | *Pseudomonadales* | *Pseudomonadaceae* | sf_1 8754^b^ | AB076857.1 | 2.2 |
|  |  | *γ-proteobacteria* | *Pseudomonadales* | *Pseudomonadaceae* | sf_1 8561 | AF332541.1 | 2.2 |
|  |  | *γ-proteobacteria* | *Pseudomonadales* | *Pseudomonadaceae* | sf_1 8687 | AF094738.1 | 2.2 |
|  |  | *γ-proteobacteria* | *Pseudomonadales* | *Pseudomonadaceae* | sf_1 9267 | AB001450.1 | 2.1 |
|  |  | *γ-proteobacteria* | *Pseudomonadales* | *Pseudomonadaceae* | sf_1 8263^b^ |  | 2.1 |
|  |  | *γ-proteobacteria* | *Pseudomonadales* | *Pseudomonadaceae* | sf_1 9228 | AJ297767.1 | 2.1 |
|  |  | *γ-proteobacteria* | *Pseudomonadales* | *Pseudomonadaceae* | sf_1 8813 | AY150184.1 | 2.1 |
|  |  | *γ-proteobacteria* | *Pseudomonadales* | *Pseudomonadaceae* | sf_1 9243 | Z76670.1 | 2.1 |
|  |  | *γ-proteobacteria* | *Pseudomonadales* | *Pseudomonadaceae* | sf_1 8338 | AF267911.1 | 2.1 |
|  |  | *γ-proteobacteria* | *Pseudomonadales* | *Pseudomonadaceae* | sf_1 8553 | AB046997.1 | 2.1 |
|  |  | *γ-proteobacteria* | *Pseudomonadales* | *Pseudomonadaceae* | sf_1 9221 | AJ492830.1 | 2.1 |

**Supplemental Table S2.** Cont.

| Treatment | Phylum | Class | Order | Family | PhyloChip ID^a^ | GenBank Accession | Fold Increase |
| --- | --- | --- | --- | --- | --- | --- | --- |
|  |  | *γ-proteobacteria* | *Pseudomonadales* | *Pseudomonadaceae* | sf_1 9240 | AJ278812.1 | 2.0 |
|  |  | *γ-proteobacteria* | *Pseudomonadales* | *Pseudomonadaceae* | sf_1 8708 |  | 2.0 |
|  |  | *γ-proteobacteria* | *Pseudomonadales* | *Pseudomonadaceae* | sf_1 9343^b^ | AF448515.1 | 2.0 |
|  |  | *γ-proteobacteria* | SAR86 | Unclassified | sf_1 8962 | AF406526.1 | 2.2 |
|  |  | *γ-proteobacteria* | *Thiotrichales* | *Piscirickettsiaceae* | sf_3 8845^b^ | AF513949.1 | 2.3 |
|  |  | *γ-proteobacteria* | Unclassified | Unclassified | sf_3 9367^b^ | AF387348.1 | 3.8 |
|  |  | *γ-proteobacteria* | Unclassified | Unclassified | sf_3 9473^b^ | AF468261.1 | 2.0 |
|  |  | *γ-proteobacteria* | *Vibrionales* | *Vibrionaceae* | sf_1 8723^b^ | AF118021.1 | 3.1 |
|  |  | *γ-proteobacteria* | *Vibrionales* | *Vibrionaceae* | sf_1 8888^b^ | AJ845014.1 | 2.3 |
|  |  | *γ-proteobacteria* | *Vibrionales* | *Vibrionaceae* | sf_1 8267^b^ | AJ491290.1 | 2.2 |
|  |  | *γ-proteobacteria* | *Vibrionales* | *Vibrionaceae* | sf_1 8665^b^ | AY257975.1 | 2.2 |
|  |  | *γ-proteobacteria* | *Vibrionales* | *Vibrionaceae* | sf_1 8798^b^ | AF172840.1 | 2.2 |
|  |  | *γ-proteobacteria* | *Vibrionales* | *Vibrionaceae* | sf_1 8999^b^ | AY292944.1 | 2.0 |
|  |  | *γ-proteobacteria* | *Xanthomonadales* | *Xanthomonadaceae* | sf_3 9150 |  | 2.3 |
| G3P (pH 6.8) | *Crenarchaeota* | *Thermoprotei* | *Desulfurococcales* | Unclassified | sfA 9741 | X99555.1 | 2.5 |
|  |  | *Thermoprotei* | Unclassified | Unclassified | sfA 10112 | EF552404.1 | 2.3 |
|  |  | Unclassified | Unclassified | Unclassified | sfA 9126 | AJ576215.1 | 3.1 |
|  | *Euryarchaeota* | *Archaeoglobi* | *Archaeoglobales* | *Archaeoglobaceae* | sfA 9182 | AB019735.1 | 3.7 |
|  |  | *Methanobacteria* | *Methanobacteriales* | MSBL1 | sfC 9702 | AY627495.1 | 4.7 |
|  |  | *Methanomicrobia* | *Methanomicrobiales* | *Methanospirillaceae* | sfA 9955 | AB175345.1 | 4.2 |
|  |  | *Methanomicrobia* | *Methanomicrobiales* | *Methanospirillaceae* | sfA 9827 | AY692060.1 | 2.3 |
|  |  | *Thermoplasmata* | *Thermoplasmatales* | Unclassified | sfA 9314^b^ | AF544224.1 | 2.2 |
|  |  | *Thermoplasmata* | *Thermoplasmatales* | *Picrophilaceae* | sfA 9358 | NC_005877.1 | 2.0 |
|  |  | *Thermoplasmata* | *Thermoplasmatales* | *Thermoplasmataceae* | sfA 10132 | AF544219.1 | 2.0 |
|  | Unclassified | Unclassified | Unclassified | Unclassified | sfA 9689^b^ | AB007304.1 | 2.8 |
|  |  | Unclassified | Unclassified | Unclassified | sfA 9553 | AY522884.1 | 2.3 |
|  |  | Unclassified | Unclassified | Unclassified | sfA 8838 | AB111479.1 | 2.5 |
|  |  | Unclassified | Unclassified | Unclassified | sfB 9261 | AY592555.1 | 2.5 |
|  | *Acidobacteria* | *Holophagae* | *Holophagales* | *Holophagaceae* | sf_14 208 | AJ519665.1 | 2.5 |
|  |  | *Chloracidobacteria* | Unclassified | Unclassified | sf_1 87 | AF097766.1 | 2.7 |
|  |  | *Chloracidobacteria* | Unclassified | Unclassified | sf_1 790 | Z95709.1 | 2.7 |
|  | *Bacteroidetes* | *Bacteroidetes* | *Bacteroidales* | *Prevotellaceae* | sf_1 6236^b^ |  | 5.7 |
|  |  | *Bacteroidetes* | *Bacteroidales* | *Prevotellaceae* | sf_1 6152^b^ | AF001768.1 | 3.3 |

**Supplemental Table S2.** Cont.

| Treatment | Phylum | Class | Order | Family | PhyloChip ID^a^ | GenBank Accession | Fold Increase |
| --- | --- | --- | --- | --- | --- | --- | --- |
|  |  | *Bacteroidetes* | Unclassified | Unclassified | sf_15 5511 | AJ535256.1 | 2.1 |
|  |  | *Flavobacteria* | *Flavobacteriales* | *Flavobacteriaceae* | sf_1 5971 | M62799.1 | 2.6 |
|  |  | *Flavobacteria* | *Flavobacteriales* | *Flavobacteriaceae* | sf_1 5991^b^ | AB032506.1 | 2.5 |
|  |  | *Flavobacteria* | *Flavobacteriales* | *Flavobacteriaceae* | sf_1 5997 | M62797.1 | 2.2 |
|  |  | *Flavobacteria* | *Flavobacteriales* | *Flavobacteriaceae* | sf_1 5942 |  | 2.2 |
|  |  | *Sphingobacteria* | *Sphingobacteriales* | Unclassified | sf_11 6148^b^ | AF255635.1 | 50.4 |
|  |  | *Sphingobacteria* | *Sphingobacteriales* | Unclassified | sf_11 5283^b^ | AF361195.1 | 13.6 |
|  |  | *Sphingobacteria* | *Sphingobacteriales* | Unclassified | sf_11 5288^b^ |  | 11.9 |
|  |  | *Sphingobacteria* | *Sphingobacteriales* | Unclassified | sf_11 5948^b^ | AF423292.1 | 10.5 |
|  |  | *Sphingobacteria* | *Sphingobacteriales* | Unclassified | sf_11 5872^b^ |  | 5.0 |
|  |  | *Sphingobacteria* | *Sphingobacteriales* | Unclassified | sf_11 6143 | AF534433.1 | 2.8 |
|  |  | *Sphingobacteria* | *Sphingobacteriales* | Unclassified | sf_11 6050 | AF527580.1 | 2.8 |
|  |  | *Sphingobacteria* | *Sphingobacteriales* | Unclassified | sf_11 5389^b^ | AY238335.1 | 2.7 |
|  |  | *Sphingobacteria* | *Sphingobacteriales* | Unclassified | sf_11 5619 | AY211072.1 | 2.7 |
|  |  | *Sphingobacteria* | *Sphingobacteriales* | Unclassified | sf_11 5832^b^ | AF368190.1 | 2.2 |
|  |  | *Sphingobacteria* | *Sphingobacteriales* | Unclassified | sf_11 5463^b^ | AB078068.1 | 2.1 |
|  |  | *Sphingobacteria* | *Sphingobacteriales* | Unclassified | sf_11 6123 | AB078055.1 | 2.0 |
|  |  | *Sphingobacteria* | *Sphingobacteriales* | Unclassified | sf_11 5642^b^ | AY218691.1 | 2.0 |
|  | *Chlamydiae* | *Chlamydiae* | *Chlamydiales* | *Chlamydiaceae* | sf_1 4820 | NC_002179.2 | 2.0 |
|  | *Cyanobacteria* | *Cyanobacteria* | *Chloroplasts* | *Chloroplasts* | sf_11 5098 | AF289245.1 | 2.2 |
|  | *Firmicutes* | *Bacilli* | *Bacillales* | *Bacillaceae* | sf_1 3900 | X68416.1 | 3.2 |
|  |  | *Bacilli* | *Bacillales* | *Bacillaceae* | sf_1 3517 | AF500007.1 | 2.2 |
|  |  | *Bacilli* | *Bacillales* | *Halobacillaceae* | sf_1 3769 | AY121437.1 | 2.0 |
|  |  | *Bacilli* | *Lactobacillales* | *Streptococcaceae* | sf_1 3722 | NC_002662.1 | 2.7 |
|  | *Proteobacteria* | *α−proteobacteria* | *Rickettsiales* | *Rickettsiaceae* | sf_3 7481 | AF179630.1 | 2.4 |
|  |  | *α−proteobacteria* | *Rickettsiales* | *Rickettsiaceae* | sf_3 6648 | AF035160.1 | 2.1 |
|  |  | *δ−proteobacteria* | *Myxococcales* | Unclassified | sf_1 10259 |  | 2.2 |
|  |  | *γ−proteobacteria* | *Alteromonadales* | *Alteromonadaceae* | sf_1 8600 | AB094412.1 | 2.0 |
|  | *Proteobacteria* | *γ−proteobacteria* | *Alteromonadales* | *Shewanellaceae* | sf_1 8662 |  | 2.1 |
|  |  | *γ−proteobacteria* | *Enterobacteriales* | *Enterobacteriaceae* | sf_6 103^b^ |  | 16.4 |
|  |  | *γ−proteobacteria* | *Enterobacteriales* | *Enterobacteriaceae* | sf_1 9293^b^ | AJ245598.1 | 2.6 |
|  |  | *γ−proteobacteria* | *Enterobacteriales* | *Enterobacteriaceae* | sf_1 9420^b^ |  | 2.5 |
|  |  | *γ−proteobacteria* | *Pseudomonadales* | *Pseudomonadaceae* | sf_1 9219 | AF181576.1 | 2.2 |

**Supplemental Table S2.** Cont.

| Treatment | Phylum | Class | Order | Family | PhyloChip ID^a^ | GenBank Accession | Fold Increase |
| --- | --- | --- | --- | --- | --- | --- | --- |
|  |  | *γ−proteobacteria* | *Pseudomonadales* | *Pseudomonadaceae* | sf_1 9343^b^ | AF448515.1 | 2.2 |
|  |  | *γ−proteobacteria* | *Pseudomonadales* | *Pseudomonadaceae* | sf_1 8344 | AF425998.1 | 2.1 |
|  |  | *γ−proteobacteria* | *Pseudomonadales* | *Pseudomonadaceae* | sf_1 8777 | AB095005.1 | 2.1 |
|  |  | *γ−proteobacteria* | *Pseudomonadales* | *Pseudomonadaceae* | sf_1 9068 | AF143245.1 | 2.0 |
|  |  | *γ−proteobacteria* | *Pseudomonadales* | *Pseudomonadaceae* | sf_1 8508 | AF530073.1 | 2.0 |
|  |  | *γ−proteobacteria* | *Pseudomonadales* | *Pseudomonadaceae* | sf_1 8852 | AF063219.1 | 2.0 |
|  |  | *γ−proteobacteria* | *Pseudomonadales* | *Pseudomonadaceae* | sf_1 9295 |  | 2.0 |
|  |  | *γ−proteobacteria* | *Pseudomonadales* | *Pseudomonadaceae* | sf_1 9238 |  | 2.0 |
|  |  | *γ−proteobacteria* | *Vibrionales* | *Vibrionaceae* | sf_1 8723^b^ | AF118021.1 | 2.1 |
|  | Unclassified | Unclassified | Unclassified | Unclassified | sf_160 539^b^ |  | 3.1 |

Fold increases in fluorescence are relative to comparable treatment pH without organophosphate

^a^Microarray 16S rRNA gene subfamily and OTU identification.

^b^OTUs undetected in soils prior to treatments.
