# Supplementary material for: Microbial Community Responses to Organophosphate Substrate Additions in Contaminated Subsurface Sediments
Source: PLoS One. 2014 Jun 20;9(6):e100383. doi: 10.1371/journal.pone.0100383 (PMC4065101; doi:10.1371/journal.pone.0100383)
Supplement: Table S3 — OTUs with a 2-fold or greater relative increase in fluorescence intensity detected in two or more treatments. (DOC) [file pone.0100383.s005.doc]

**Supplemental Table S3.** OTUs with a 2-fold or greater relative increase in fluorescence intensity detected in two or more treatments.

| Treatments | Phylum | Class | | Order | Family | PhyloChip ID^a^ | GenBank Accession |
| --- | --- | --- | --- | --- | --- | --- | --- |
| G3P (pH 5.5)  G2P (pH 6.8)  G3P (pH 6.8) | *Euryarchaeota* | *Methanobacteria* | | *Methanobacteriales* | MSBL1 | sfC 9702 | AY627495.1 |
| G3P (pH 5.5)  G2P (pH 6.8) | *Crenarchaeota* | Unclassified | | Unclassified | Unclassified | sfA 9131 | AY592502.1 |
|  | *Deferribacteres* | *Deferribacteres* | | Unclassified | Unclassified | sf_1 797 | AJ515881.1 |
| G2P (pH 6.8)  G3P (pH 6.8) | *Crenarchaeota* | Unclassified | | Unclassified | Unclassified | sfA 9126 | AJ576215.1 |
|  | *Acidobacteria* | *Holophagae* | | *Holophagales* | *Holophagaceae* | sf_14 208 | AJ519665.1 |
|  |  | *Chloracidobacteria* | | Unclassified | Unclassified | sf_1 790 | Z95709.1 |
|  | *Bacteroidetes* | *Bacteroidetes* | | *Bacteroidales* | *Prevotellaceae* | sf_1 6152^b^ | AF001768.1 |
|  |  | *Bacteroidetes* | | *Bacteroidales* | *Prevotellaceae* | sf_1 6236^b^ |  |
|  |  | *Sphingobacteria* | | *Sphingobacteriales* | Unclassified | sf_11 6050^b^ | AF527580.1 |
|  | *Proteobacteria* | γ*-proteobacteria* | | *Enterobacteriales* | *Enterobacteriaceae* | sf_6 103^b^ |  |
|  | Unclassified | Unclassified | | Unclassified | Unclassified | sf_160 539 |  |
| G3P (pH 5.5)  G3P (pH 6.8) | *Euryarchaeota* | | *Archaeoglobi* | *Archaeoglobales* | *Archaeoglobaceae* | sfA 9182 | AB019735.1 |
|  | *Firmicutes* | | *Bacilli* | *Bacillales* | *Bacillaceae* | sf_1 3900 | X68416.1 |
|  | *Proteobacteria* | | *γ-proteobacteria* | *Alteromonadales* | *Shewanellaceae* | sf_1 8662 |  |
|  |  | | *γ-proteobacteria* | *Enterobacteriales* | *Enterobacteriaceae* | sf_1 9420^b^ |  |
|  |  | | *γ-proteobacteria* | *Enterobacteriales* | *Enterobacteriaceae* | sf_1 9293^b^ | AJ245598.1 |
|  |  | | *γ-proteobacteria* | *Pseudomonadales* | *Pseudomonadaceae* | sf_1 9238 |  |
|  |  | | *γ-proteobacteria* | *Pseudomonadales* | *Pseudomonadaceae* | sf_1 9295 |  |
|  |  | | *γ-proteobacteria* | *Pseudomonadales* | *Pseudomonadaceae* | sf_1 8344 | AF425998.1 |
|  |  | | *γ-proteobacteria* | *Pseudomonadales* | *Pseudomonadaceae* | sf_1 8777 | AB095005.1 |
|  |  | | *γ-proteobacteria* | *Pseudomonadales* | *Pseudomonadaceae* | sf_1 9068 | AF143245.1 |
|  |  | | *γ-proteobacteria* | *Pseudomonadales* | *Pseudomonadaceae* | sf_1 8852 | AF063219.1 |
|  |  | | *γ-proteobacteria* | *Pseudomonadales* | *Pseudomonadaceae* | sf_1 9343^b^ | AF448515.1 |
|  |  | | *γ-proteobacteria* | *Pseudomonadales* | *Pseudomonadaceae* | sf_1 9219 | AF181576.1 |
|  |  | | *γ-proteobacteria* | *Vibrionales* | *Vibrionaceae* | sf_1 8723^b^ | AF118021.1 |

**Supplemental Table S3.** Cont.

| Treatments | Phylum | Class | Order | | Family | | | PhyloChip ID^a^ | | GenBank Accession |
| --- | --- | --- | --- | --- | --- | --- | --- | --- | --- | --- |
| G2P (pH 5.5)  G3P (pH 5.5) | *Proteobacteria* | *α-proteobacteria* | *Rhizobiales* | | *Methylocystaceae* | | | sf_1 7255 | | AB159685.1 |
|  |  | *α-proteobacteria* | *Rhodospirillales* | | *Rhodospirillaceae* | | | sf_1 7109^b^ | | AY189753.1 |
|  |  | *β-proteobacteria* | *Burkholderiales* | | *Comamonadaceae* | | | sf_1 7834^b^ | | AB021418.1 |
|  |  | *β-proteobacteria* | *Burkholderiales* | | *Comamonadaceae* | | | sf_1 7882 | | AF526937.1 |
|  |  | *γ-proteobacteria* | *Enterobacteriales* | | *Enterobacteriaceae* | | | sf_1 8758^b^ | | U80201.1 |
|  |  | *γ-proteobacteria* | *Thiotrichales* | | *Piscirickettsiaceae* | | | sf_3 8845^b^ | | AF513949.1 |
|  |  | *γ-proteobacteria* | *Xanthomonadales* | | *Xanthomonadaceae* | | | sf_3 9150 | |  |
| G2P (pH 5.5)  G2P (pH 6.8) | *Nitrospira* | *Nitrospira* | *Nitrospirales* | | *Nitrospiraceae* | | sf_1 864 | | | Y14644.1 |
|  | *Proteobacteria* | *α-proteobacteria* | *Rhizobiales* | | *Rhizobiaceae* | | sf_1 6847^b^ | | | U29387.1 |
|  |  | *α-proteobacteria* | *Rhizobiales* | | *Phyllobacteriaceae* | | sf_1 6876^b^ | | | AF003376.1 |
|  |  | *α-proteobacteria* | *Rhizobiales* | | *Phyllobacteriaceae* | | sf_1 6962 | | | AJ132378.1 |
|  |  | *α-proteobacteria* | *Rhizobiales* | | *Rhizobiaceae* | | sf_1 6725^b^ | | | AL591782.1 |
|  |  | *α-proteobacteria* | *Rhizobiales* | | *Rhizobiaceae* | | sf_1 7380^b^ | | | AB118158.1 |
| G2P (pH 5.5)  G3P (pH 5.5)  G2P (pH 6.8) | *Proteobacteria* | *α-proteobacteria* | *Rhizobiales* | *Phyllobacteriaceae* | | sf_1 6854^b^ | | |  | |
| G2P (pH 5.5)  G3P (pH 5.5)  G3P (pH 6.8) | *Proteobacteria* | *γ-proteobacteria* | *Pseudomonadales* | *Pseudomonadaceae* | | sf_1 8508 | | | AF530073.1 | |

^a^Microarray 16S rRNA gene subfamily and OTU identification.

^b^OTUs undetected in soils prior to treatments.
